# A novel unconventional T cell population enriched in Crohn’s disease

**DOI:** 10.1136/gutjnl-2021-325373

**Published:** 2022-03-09

**Authors:** Elisa Rosati, Gabriela Rios Martini, Mikhail V Pogorelyy, Anastasia A Minervina, Frauke Degenhardt, Mareike Wendorff, Soner Sari, Gabriele Mayr, Antonella Fazio, Christel Marie Dowds, Charlotte Hauser, Florian Tran, Witigo von Schönfels, Julius Pochhammer, Maria A Salnikova, Charlot Jaeckel, Johannes Boy Gigla, Sanaz Sedghpour Sabet, Matthias Hübenthal, Esther Schiminsky, Stefan Schreiber, Philip C Rosenstiel, Alexander Scheffold, Paul G Thomas, Wolfgang Lieb, Bernd Bokemeyer, Maria Witte, Konrad Aden, Alexander Hendricks, Clemens Schafmayer, Jan-Hendrick Egberts, Ilgar Z Mamedov, Petra Bacher, Andre Franke

**Affiliations:** 1 Institute of Clinical Molecular Biology, Christian-Albrechts University of Kiel, Kiel, Schleswig-Holstein, Germany; 2 Institute of Immunology, Christian-Albrechts University of Kiel, Kiel, Schleswig-Holstein, Germany; 3 Shemyakin-Ovchinnikov Institute of Bioorganic Chemistry, Russian Academy of Sciences, Moscow, Russian Federation; 4 Department of Immunology, St Jude Children's Research Hospital, Memphis, Tennessee, USA; 5 Department of Visceral and Thoracic Surgery, Universitatsklinikum Schleswig-Holstein, Kiel, Schleswig-Holstein, Germany; 6 Department of Internal Medicine I, Universitätsklinikum Schleswig-Holstein, Kiel, Schleswig-Holstein, Germany; 7 Department of Dermatology, University Hospital Schleswig Holstein, Kiel, Schleswig-Holstein, Germany; 8 Institute of Epidemiology and Biobank POPGEN, Christian-Albrechts-University of Kiel, Kiel, Germany; 9 Interdisciplinary Crohn Colitis Centre Minden, Minden, Germany; 10 Department of General Surgery, Rostock University Medical Center, Rostock, Mecklenburg-Vorpommern, Germany; 11 CEITEC, Masaryk University, Brno, Czech Republic; 12 Dmitry Rogachev National Research Center of Pediatric Hematology, Moscow, Russian Federation; 13 Center for Precision Genome Editing and Genetic Technologies for Biomedicine, Moscow, Russian Federation

**Keywords:** T-cell receptor, alpha beta T cells, Crohn's disease, mucosal immunology, IBD

## Abstract

**Objective:**

One of the current hypotheses to explain the proinflammatory immune response in IBD is a dysregulated T cell reaction to yet unknown intestinal antigens. As such, it may be possible to identify disease-associated T cell clonotypes by analysing the peripheral and intestinal T-cell receptor (TCR) repertoire of patients with IBD and controls.

**Design:**

We performed bulk TCR repertoire profiling of both the TCR alpha and beta chains using high-throughput sequencing in peripheral blood samples of a total of 244 patients with IBD and healthy controls as well as from matched blood and intestinal tissue of 59 patients with IBD and disease controls. We further characterised specific T cell clonotypes via single-cell RNAseq.

**Results:**

We identified a group of clonotypes, characterised by semi-invariant TCR alpha chains, to be significantly enriched in the blood of patients with Crohn’s disease (CD) and particularly expanded in the CD8^+^ T cell population. Single-cell RNAseq data showed an innate-like phenotype of these cells, with a comparable gene expression to unconventional T cells such as mucosal associated invariant T and natural killer T (NKT) cells, but with distinct TCRs.

**Conclusions:**

We identified and characterised a subpopulation of unconventional Crohn-associated invariant T (CAIT) cells. Multiple evidence suggests these cells to be part of the NKT type II population. The potential implications of this population for CD or a subset thereof remain to be elucidated, and the immunophenotype and antigen reactivity of CAIT cells need further investigations in future studies.

**Significance of this study**

**What is already known on this subject?**

- T lymphocytes are known to drive Crohn’s disease (CD) pathogenesis and progression.
- Previous studies identified T-cell receptor (TCR) repertoire traits and specific clonotypes associated with disease status and prognosis.
- Interindividual variability challenged the identification and validation of disease-associated TCRs shared among multiple patients.

**What are the new findings?**

- T cells with a semi-invariant TCRα motif were strongly enriched in the blood of patients with CD.
- These clonotypes are mostly found among CD8+ T cells and have an unconventional T cell phenotype.
- Multiple evidence suggests these cells to be part of the natural killer T (NKT) type II population.

**How might it impact on clinical practice in the foreseeable future?**

- The identified clonotypes draw attention on the possible role of NKT type II cells in CD and deserve further investigation.
- These clonotypes may discriminate CD from other forms of IBD, as well as other diseases and provide a new biomarker for CD.

## Introduction

Dysregulated T cell reactions against intestinal antigens are considered to be causal or driving factors for IBD.^1 2^ As hypothesised also for other inflammatory and autoimmune diseases, for example, multiple sclerosis^3^ or type 1 diabetes,^4^ the excessive immune response in IBD may be directed against common, yet still unknown, disease-driving antigens. In genetically predisposed hosts, antigens derived from a dysbiotic microbiome may come into contact with T cells through the impaired intestinal epithelial barrier, which may lead to uncontrolled and dysregulated immune responses.^2^ Accordingly, it may be possible to identify specific disease-associated T cells within the peripheral blood and/or at the intestinal inflammation site of patients with IBD.

Although the two main forms of IBD, namely Crohn’s disease (CD) and UC, are often referred to as generally ‘IBD’, they are clinically two independent diseases with different disease phenotypes but partially overlapping genotypes.^5^ CD may localise in any part of the GI tract, mostly in the small intestine, while UC develops only in the colon.^6^ Additionally, while both diseases are thought to be mediated by dysregulated T cell responses, different T cell subsets are hypothesised to play a role in disease pathogenesis and progression.^7^ Phenotypes and markers distinguishing the two diseases exist but are limited, sometimes resulting in incorrect diagnoses. Further insights differentiating the two forms of IBD could result in a better understanding of the pathophysiology, as well as the development of future strategies for diagnosis and therapy of the diseases.

Most T cells are characterised by a unique T-cell receptor (TCR), formed by an alpha and beta chain. T cells recognise antigenic peptides presented by the major histocompatibility complex (MHC). On recognition of an antigen via the TCR, T cells may expand clonally, resulting in a population of cells with identical TCRs (‘clonotypes’). The TCR repertoire, defined as the collection of unique TCRs of an individual, is extremely variable, with up to 10^11^ unique clonotypes present in a single person.^8^ Therefore, the composition of the TCR repertoire, that is, the diversity of different TCRs or the expansion of individual clonotypes, could provide important insights into disease-associated alterations of the T cell reaction.

TCR repertoire profiling using high-throughput sequencing has emerged as a method for the analysis of a variety of diseases and conditions.^9^ The identification of disease-associated T cell clonotypes in patients with IBD could be an important step towards elucidating how changes in the TCR repertoire impact the diseases, towards the identification of pathogenic T cells and antigenic triggers, and towards the development of new diagnostic and therapeutic strategies. Previous studies identified expanded clonotypes in IBD intestinal samples as compared with blood samples^9–12^ and correlated the presence of patient-specific TCRs in the intestinal tissue with disease recurrence after surgery.^12^ However, up to now, the identification of disease-associated clonotypes in multiple individuals has proven to be challenging due to the interindividual variability of the diseases as well as of the TCR repertoire. Further, the acquisition of intestinal material is often a limiting factor.^9 10 12^ Moreover, most studies look only at the TCR beta repertoire, which is more variable and considered more informative to infer unique clonotypes as well as antigen and HLA specificity. As a result, the TCR alpha repertoire is greatly understudied. Only very few datasets, none of which includes IBD, are available for both alpha and beta chains.

A subset of T cells is formed by the so-called unconventional T cells, which recognise non-classical HLA molecules. The most known unconventional T cells include mucosal associated invariant T (MAIT) cells which bind to MR1, and natural killer T (NKT) cells which bind to the MHC-like molecule CD1d. MAIT cells and NKT type I or invariant NKT (iNKT) cells are characterised by a semi-invariant TCR alpha repertoire formed by the *TRAV1-2*, *TRAJ33/20/12* and *TRAV10*, *TRAJ18* genes, respectively.^13–16^ In contrast, NKT type II cells express a polyclonal repertoire. Although there are still many open questions about unconventional T cells, they have been described to recognise ligands of microbial origin and thus to interact with the intestinal microbiota.^15^ While MAIT cells are known to be decreased in blood of patients with IBD and to accumulate in the inflamed intestinal mucosa,^17^ the role of NKT type II cells in IBD is unclear, with contradictory evidences on the protective or pathogenic role of these cells.^18^

Here, we performed an integrated analysis of multiple independent sample collections for which both alpha and beta TCR repertoire profiling, and HLA data were generated. We detected a selective enrichment of specific T cell clonotypes in the blood of patients with CD, which was further characterised by single-cell RNAseq and TCRseq. The herein identified clonotypes are characterised by a semi-invariant motif of the TCR alpha chain, as well as shared gene expression (GEX) markers with unconventional T cells. Based on our own results and on works by others,^19^ we hypothesise these cells to be a new specific subgroup of NKT type II cells, whose enrichment in patients with CD we show for the first time.

**Figure 1:**
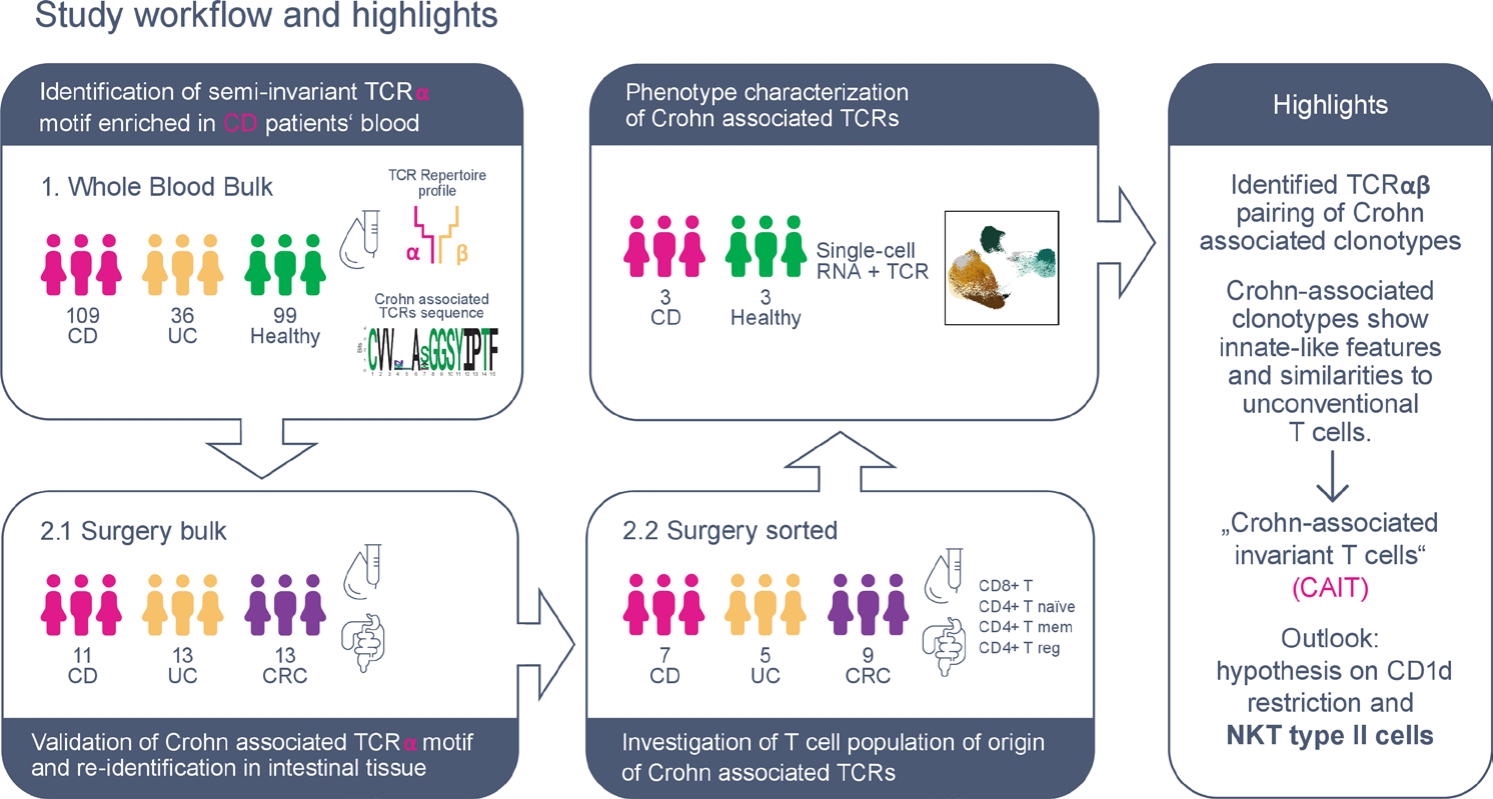
Graphical abstract of study workflow and main results. Two sample collections were used for T-cell receptor (TCR) repertoire profiling of patients with IBD and controls: (1) the whole blood collection included bulk blood samples from IBD and healthy controls; the surgery collection included matched blood, intestinal tissue and intestinal lymph nodes of patients with IBD and disease control (colon cancer, CRC), both from (2.1) bulk tissue and (2.2) sorted T cell populations. Multiple TCRs, sharing a semi-invariant TCR alpha motif, were identified to be enriched in patients with CD and particularly in the CD8^+^ fraction. Cells carrying this TCR motif were reidentified in single-cell RNA and TCR data of three recontacted patients with CD and three age-matched and sex-matched healthy controls. Single-cell gene expression analysis revealed that CD-associated clonotypes have an innate-like phenotype enriched in CD8^+^ T cells and comparable to unconventional T cells, that is, mucosal associated invariant T (MAIT) and natural killer T (NKT) cells. Thus, for simplicity, we refer to these clonotypes as Crohn-associated invariant T (CAIT) cells throughout the manuscript. Finally, based on our own result as well as other studies, we hypothesise CAIT clonotypes to be part of NKT type II family and reactive to the CD1d HLA-like molecule.

A summary of the performed analyses and sample collections is presented in figure 1 and online supplemental table S1.

10.1136/gutjnl-2021-325373.supp1 Supplementary data

## Results

### Identification of TCRs enriched or depleted in the blood of patients with CD

We employed a prospective blood sample collection of 109 CD, 36 patients with UC and 99 healthy controls (online supplemental table S2) with the aim of identifying specific T cell clonotypes associated with CD. Thus, we performed TCR repertoire profiling of the TCR alpha chain and TCR beta chain.^20^ Summary of the analysed bulk TCR data can be found in online supplemental table S3.

10.1136/gutjnl-2021-325373.supp2 Supplementary data

**Figure 2:**
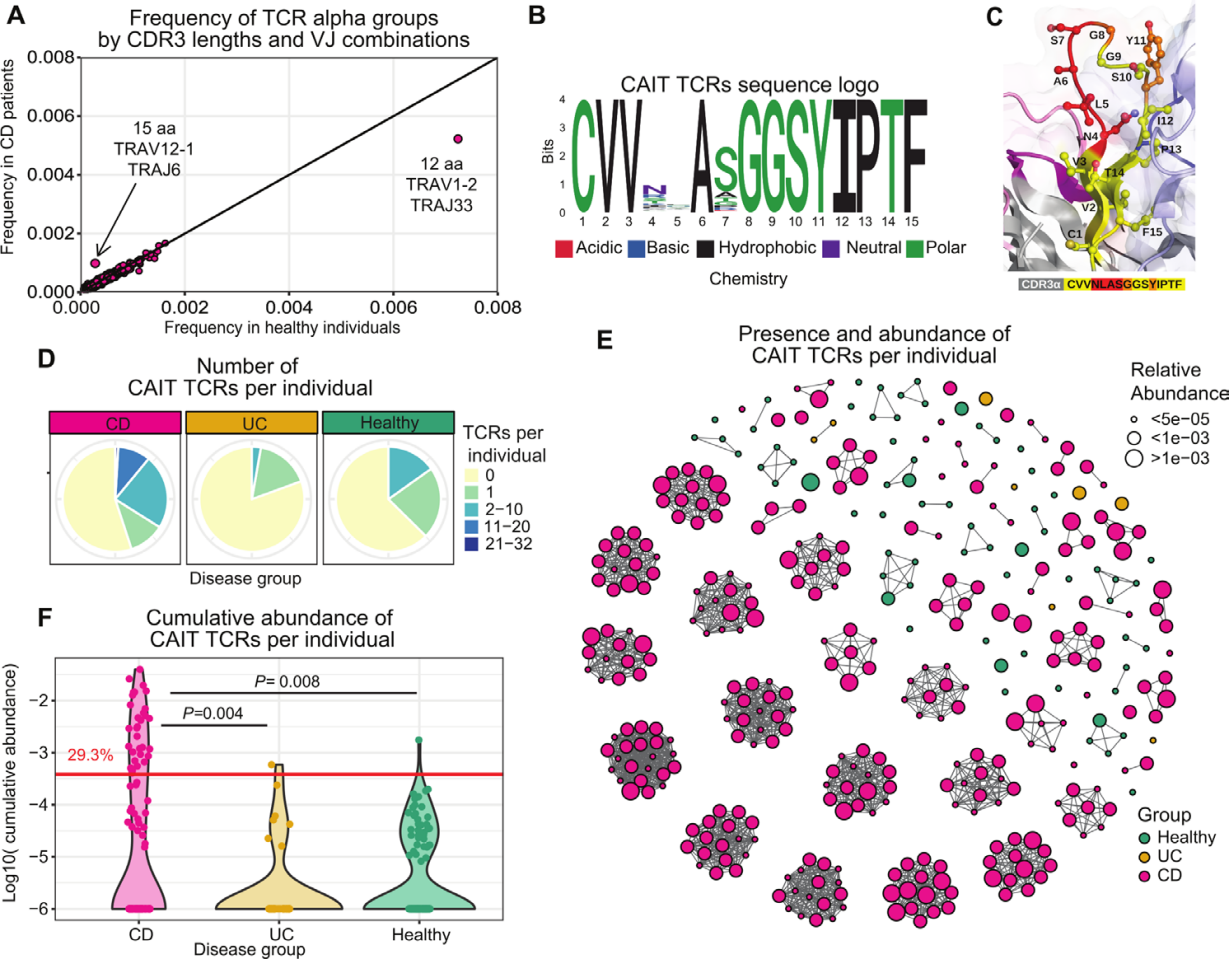
Identification of Crohn-associated invariant T (CAIT) alpha chains in blood. (A) Scatterplot of the frequency of all CDR3 lengths and VJ gene combinations in patients with Crohn’s disease (CD) and healthy controls. Enrichment of 15 aa long TRAV12-1_TRAJ6 clonotypes and depletion of 12 aa long TRAV1-2_TRAJ33 clonotypes was observed in patients with CD. (B) Logo plot of the TRAV12-1_TRAJ6 CDR3 amino acid motif enriched in CD. T-cell receptors (TCRs) with this motif are hereafter referred to as ‘Crohn-associated TCRs’ and define CAIT cells. (C) Zoom-in of CDR3α positions 1–15 (CVVNLASGGSYIPTF) depicting amino acid positions as ball-and-stick and coloured as indicated in the sequence panel below. Amino acid positions 4–7 (NLAS) are coloured in red. In orange, amino acid positions G8 and Y11, that are predicted to directly interact with the epitope together with 6A and 7S due to the orientation of their side chains towards the surface. (D) Pie chart showing the proportion of individuals carrying different numbers of CD-associated clonotypes. (E) Network plot showing enrichment of the specific sequences in CD. Only samples where CAIT clonotypes were present are shown. Each separated group of nodes/cluster represents one individual. Each node represents one CAIT TCR of the (motif group of figure 2B). The size of the node reflects the abundance of the clonotype in a specific sample/individual. Colour of the nodes represents the disease group. It should be noted that the same TCR may be present multiple times in the plot if it is found in multiple individuals. (F) Log-transformed cumulative abundance of CD-associated clonotypes per individual. An abundance of 0 is represented as −6 on the log scale. In patients above the horizontal red line, CAIT clonotypes account for more than 2.5% of the whole blood repertoire, in detail 32/109 tested patients with CD (29.3%) but in only 1/36 (2.7%) patients with UC and 1/99 (1%) healthy individuals. Differences between disease groups have been assessed using Mann-Whitney U test, followed by false discovery rate (FDR) multiple comparison correction.

First, we investigated whether we could identify specific clonotypes that were selectively enriched or depleted in the blood of patients with CD. As a first exploratory analysis, we compared the frequency of TCR alpha and TCR beta clonotypes grouped in TCR families by the variable (V) gene, the joining (J) gene, and CDR3 region length (in amino acids) in patients with CD versus patients with UC and versus healthy controls. Results showed no differences for TCR beta chains (online supplemental figure S1A, B). For TCR alpha chains, however, clonotypes carrying a CDR3 sequence of 12 amino acids and gene combination *TRAV1-2*_*TRAJ33* were less frequent in patients with CD as compared with healthy individuals and patients with UC (figure 2A and online supplemental figure S1C). This was confirmed via Fisher’s exact test of single TCR alpha sequences of this group (p values in online supplemental table S3B). These CDR3 length and gene combination have previously been annotated as MAIT cells.^14^ In fact, MAIT cells have been shown to be decreased in the blood of patients with IBD.^21 22^

In patients with CD, a group of TCR alpha chains defined by a 15 amino acids CDR3 region and with the gene combination *TRAV12-1*_*TRAJ6* were enriched as compared with healthy controls or patients with UC (figure 2A and online supplemental figure S1C). The Fisher’s exact test confirmed that >10 clonotypes of the *TRAV12-1*_*TRAJ6* group were found in a significantly higher number of patients with CD than controls (p values in online supplemental table S3B). Clonotypes of this TCR group, identified as significantly enriched in CD, share the semi-invariant CDR3 motif CVV**A*GGSYIPTF (figure 2B). For simplicity, throughout the manuscript, we refer to these clonotypes as Crohn associated invariant T (CAIT) clonotypes.

While positions 4 and 5 of the identified CDR3 motif are the most variable, positions 6 and 7 are more conserved and occupied by A and mostly S amino acids respectively. Moreover, the predicted 3D structure of the TCR alpha CDR3 loop showed that positions 4 and 5 are mostly buried inside the TCR protein structure and may indirectly influence the loop conformation. Positions 6 and 7 are exposed and, together with positions 8 and 11, are predicted to be the most involved into epitope interaction (figure 2C and online supplemental figure S1D). However, CDR3 loops are very flexible and may adapt to different conformations when interacting with different epitopes. Thus, in principle, all CDR3 amino acid positions, excluding the terminal 3–4 residues, may contact the antigen and influence binding affinity.

A higher number of different CAIT clonotypes (different CDR3 regions) were found in patients with CD as compared with healthy controls (figure 2D). In addition, they were also more expanded in the blood of patients with CD (figure 2E) and their cumulative abundance (the sum of their relative abundance in each sample) was accordingly significantly higher in patients with CD as compared with controls (p value=0.008, figure 2F). All in all, at least one CAIT clonotype was present in 49/109 (45%) patients with CD, 7/36 (19%) patients with UC and 37/99 (37%) healthy controls. In 32/109 (29.3%) patients with CD, CAIT TCRs accounted for more than 2.5% of the whole blood repertoire, while this was the case for only 2.7% of patients with UC and 1% of healthy individuals (figure 2F). In contrast, and as previously observed in other studies, clonotypes associated with MAIT cells showed opposite behaviour and were decreased in CD (p value=3.7*10^−16^, online supplemental figure S1E, F).

To further characterise the CAIT clonotypes, we investigated a potential HLA specificity of these TCRs, which would also be an important step towards the identification of antigens against which these TCRs may be reactive to. Furthermore, certain HLA alleles are known to be associated with CD.^23^ Accordingly, it is of interest to investigate whether TCRs associated with CD may bind one of them.^24^ We genotyped our patients and imputed HLA alleles using previously published reference datasets and methods.^25 26^ No significant association was observed between the presence or abundance of CAIT clonotypes and specific HLA alleles of class I (-A, -B, -C) or class II (-DP, -DQ, -DR) (online supplemental table S4A, B). We also investigated whether CAIT TCRs were associated with phenotypic or clinical traits. No significant association was detected between the cumulative abundance or the number of CAIT TCRs, and clinical parameters such as sex, age, smoking behaviour, disease location, disease severity or previous treatment for CD (online supplemental figure S2A-N and online supplemental table S4C). Samples from patients with CD were collected prior to start of treatment with anti-TNF biologics. A non-significant trend was observed, showing increased numbers of CAIT clonotypes in patients non-responding to anti-TNF therapy (p value=0.089, online supplemental figure S2N and online supplemental table S4D). Although not reaching statistical significance, in patients affected by ileal or ileocolonic CD, CAIT TCRs were more abundant as compared with colonic CD (p value=0.07, online supplemental figure S2A and online supplemental table S4C), which may be a potential explanation for the identification of CAIT cells in only a subset of patients.

10.1136/gutjnl-2021-325373.supp3 Supplementary data

### CAIT clonotypes are present in both blood and intestinal tissue of patients with CD

**Figure 3:**
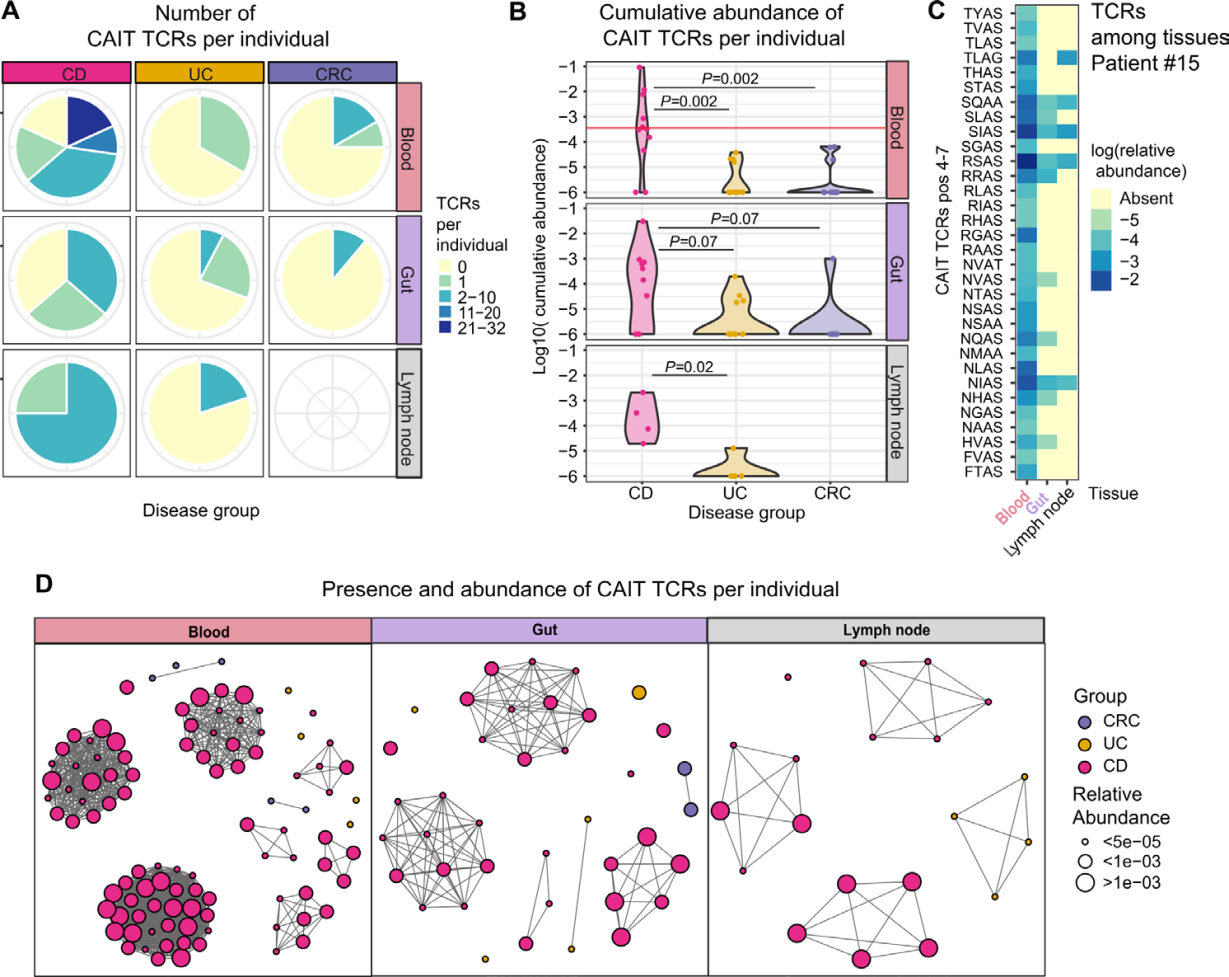
CAIT T-cell receptor (TCR) alpha chains in blood, intestinal tissue and intestinal lymph node. (A) Pie chart showing the proportion of individuals (11 Crohn’s disease (CD), 13 UC, 13 colon cancer (CRC)) carrying different numbers of Crohn-associated invariant T (CAIT) clonotypes in blood, gut and, for UC and CD only, intestinal lymph node. (B) Log-transformed cumulative abundance of Crohn-associated clonotypes per individual and tissue. In patients above the horizontal red line, CAIT clonotypes account for more than 2.5% of the whole blood repertoire (7/11 (63.6%) patients with CD, 0% patients with UC and patients with CRC). Differences between disease groups have been assessed using Mann-Whitney U test, followed by FDR multiple comparison correction. (C) Heatmap of the log-transformed relative abundance of CAIT TCRs among the tissues (x-axis) of one representative patient with CD (#15). Labels on the y-axis indicate the variable positions^4–7^ of the CDR3 sequence of CAIT TCRs. (D) Network plot showing enrichment of CAIT sequences in CD among the three analysed tissue samples. Each separated cluster thus represents one individual/sample. Each node is one TCR of the motif group of figure 1B. The size of the node expresses the abundance of the clonotype in a specific sample/individual. Colour of the vertices represents the disease group. Of note, the same TCR may be present multiple times in the plot if it is found in multiple individuals.

To verify the presence and abundance of CAIT TCRs in CD, we analysed an independent sample collection (surgery bulk in figure 1) including matched blood and intestinal tissue of 37 individuals undergoing bowel resection surgery, which was composed of 11 patients with CD, 13 patients with UC and 13 patients with colon cancer (CRC) as disease controls (online supplemental table S5). In line with the Whole Blood Bulk dataset, CAIT TCRs were confirmed to be increased in number (figure 3A) and cumulative abundance in the blood of patients with CD (p value=0.002, figure 3B–D). CAIT clonotypes were observed in the blood of 9/11 (82%) patients with CD, 4/13 (31%) patients with UC and 3/13 (23%) patients with CRC. In 7/11 (63.6%) patients with CD, CAIT TCRs composed more than 2.5% of the whole blood repertoire, while they composed maximum 0.3% and 0.6% of the blood repertoire in patients with UC and patients with CRC, respectively. The same non-significant trend was observed in intestinal tissue (p value=0.07 after multiple testing correction). For a subset of patients (4 CD and 5 UC), intestinal mesenteric lymph nodes resected together with the inflamed intestinal tissue were also analysed. CAIT clonotypes were significantly more abundant in the intestinal lymph nodes of patients with CD as compared with patients with UC (p value=0.02, figure 3A–D). While most CAIT TCRs were observed in the blood, few CAIT clonotypes were also observed to be present in multiple analysed tissues of the same individual at the same time (figure 3C).

**Figure 4:**
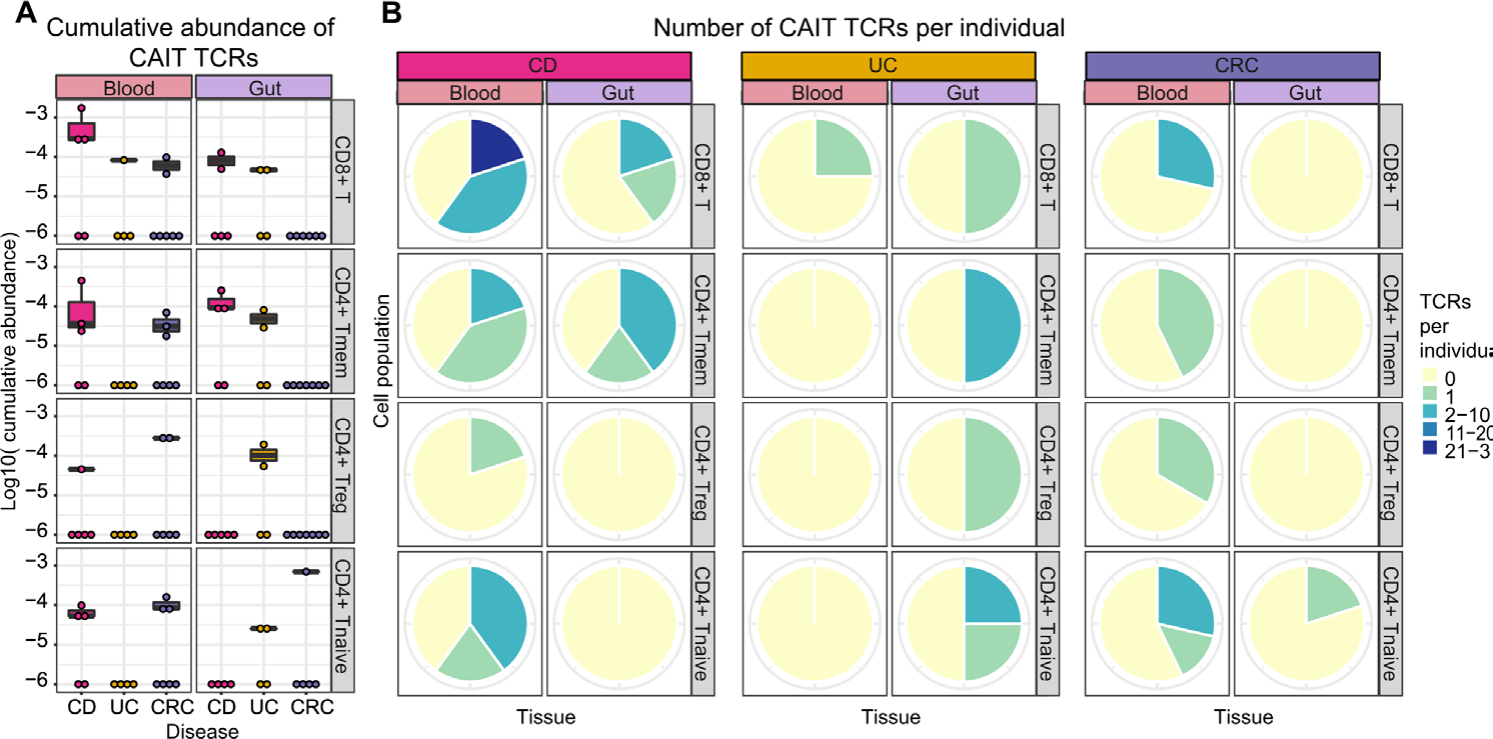
Crohn-associated invariant T (CAIT) T-cell receptor (TCR) alpha chains in sorted T cell populations. (A) Presence and log10-transformed relative abundance of CAIT TCRs (y-axis) in each individual among sorted T cell populations and analysed tissues. Particularly in CD8^+^ (first panel from top) and CD4^+^ Tmem (second panel from top), more CAIT TCRs are found in patients with Crohn’s disease (CD). (B) Pie chart showing the proportion of individuals, separated by disease group (CD: left, UC: middle, colon cancer (CRC): right) carrying different numbers of Crohn-associated clonotypes in both tissues and four cell populations. Seven patients with CD, five patients with UC and nine patients with CRC were analysed.

To answer the question whether CAIT cells occur within a particular T cell compartment, blood and intestinal T cells were sorted in CD4^+^ naïve, CD4^+^ conventional memory, CD4^+^ regulatory and CD8^+^ T cells for additional 7 patients with CD, 5 patients with UC and 9 patients with CRC (online supplemental tables S1 and S6). Surprisingly, CAIT TCRs were found in all analysed fractions, but at different frequencies. CAIT cells were most abundant in the CD8^+^ fraction, particularly in patients with CD, with up to 22 CAIT TCRs detected per individual and higher cumulative abundance as compared with the other cell compartments (figure 4A, B). In contrast, CAIT sequences were almost absent in the blood of patients with UC and higher abundance of CAIT sequences in patients with CRC was mostly found in T_reg_ and T_Naïve_ fractions.

### Beta chains paired with CAIT alpha chains are private of the single individual and show preferential V gene usage

For three patients with CD with a high proportion of CAIT cells, we next FACS-purified the CD4^+^ and CD8^+^ memory fractions and performed single-cell RNA GEX and TCR sequencing using 10× Genomics’ Chromium technology. The same analysis was performed for three sex-matched and age-matched healthy controls.

**Figure 5:**
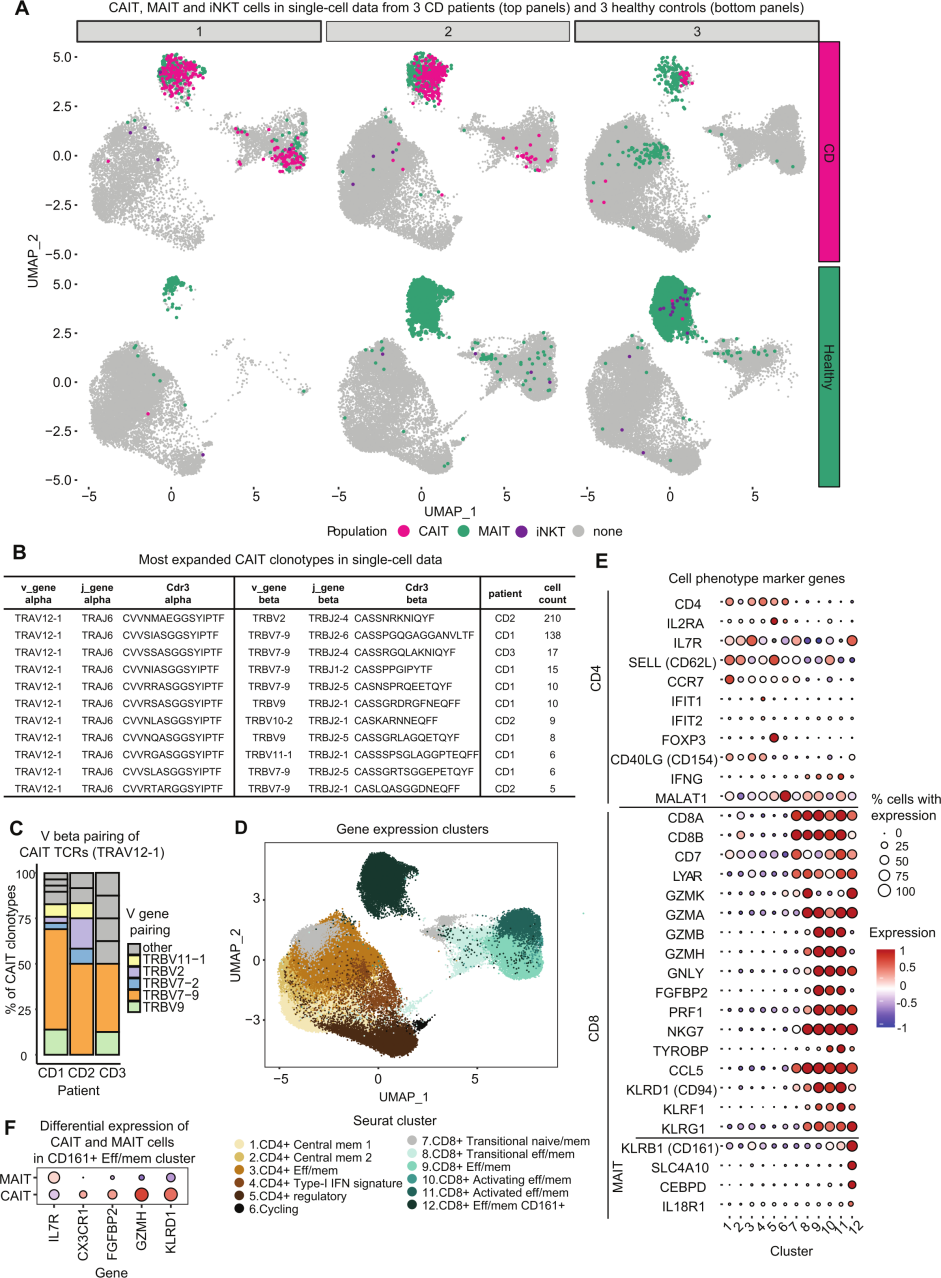
Single-cell analysis of sorted memory CD4 and CD8 cells from three patients with Crohn’s disease (CD) and three matched healthy controls. (A) Uniform Manifold Approximation and Projection (UMAP) (co-)localisation of mucosal associated invariant T (MAIT) (green), invariant NKT (iNKT) (violet) and Crohn-associated invariant T (CAIT) (pink) cells. MAIT cells are abundant in both patients with CD (top 3 panels) and healthy controls (bottom 3 panels), while CAIT cells are abundant in patients with CD but very sparse in healthy controls, only 1 (bottom panel left) and 2 (bottom panel right) CAIT cells were found in healthy controls respectively. (B) Paired T-cell receptor (TCR) alpha and beta chains of the most expanded CAIT clonotypes observed in three patients with CD through single-cell TCR analysis. (C) V genes of TCR beta chain pairing for CAIT in the three patients with CD analysed. Plotted as the proportion of unique clonotypes with a certain V gene beta pairing. (D) UMAP single-cell visualisation of Seurat functional clusters. (E) Bubble plot for Seurat cluster marker genes defining the CD4^+^ and CD8^+^ different populations. (F) Differentially expressed genes between MAIT and CAIT cells of the same function cluster (cluster 12, effector memory CD8^+^ CD161^+^ T cells).

In accordance with our initial findings, CAIT cells were identified at high abundance (22–238 CAIT cells per patient) in the three patients with CD through their TCRs (figure 5A, B and online supplemental table S7A), while they were rare in healthy controls (0–2 CAIT cells per healthy individual, figure 5A). Analysis of TCR alpha/beta pairs showed diverse TCR beta chains associated with the semi-invariant CAIT alpha sequences (figure 5B and online supplemental table S7B). Analysis of the TCR alpha/beta chain pairing in CAIT clonotypes, showed a preferential pairing (38%**–**55% of CAIT clonotypes) with beta chains carrying the *TRBV7-9* gene in all three patients (figure 5C). None of the identified paired TCR beta chains were found in any other individual of the previously analysed bulk TCR datasets (244 whole blood and 59 surgery samples). Thus, these TCR beta sequences seem to be private to single individuals or very rare in the examined population (online supplemental table S7C).

10.1136/gutjnl-2021-325373.supp4 Supplementary data

### CAIT clonotypes show an innate-like GEX profile

Based on Seurat clustering of GEX analysis,^27^ we defined 12 T cell populations of the sorted CD4^+^ and CD8^+^ cells, which are shown in figure 5D. Marker genes for each cluster are listed in online supplemental table S7D and summarised in figure 5E. CAIT cells were found mostly in the CD8^+^ fraction, more precisely in effector memory clusters characterised by high expression of *KLRD1* (CD94) and *KLRB1* (CD161), respectively, which are known markers for innate-like T and natural-killer cells (figure 5A–E and online supplemental figure S3A). Most CAIT cells were found in the CD8^+^ effector memory CD161^+^ cluster, which includes known unconventional T cell subtypes such as MAIT and NKT cells. The cluster was also characterised by upregulation of genes such as *SLC4A10*, *CEBDP* and *IL18R* that are typical GEX markers of MAIT cells^28 29^ (figure 5E and online supplemental figure S3A, B). In fact, MAIT and iNKT cells were identified in this cluster through their TCRs^30 31^ (figure 5A). A smaller subset of CAIT, MAIT and iNKT cells was also found in the CD8 effector memory cluster mostly characterised by expression of *KLRD1*, encoding for CD94, which is another marker of innate immunity and present in both NK cells, NKT cells and subsets of MAIT cells.^32 33^ Thus, CAIT cells showed an innate-like GEX profile comparable to known unconventional T cells. Moreover, their abundance in these patients was comparable to MAIT cells, while iNKT cells were very rare. To further investigate phenotypic differences between CAIT and MAIT cells, we run a differential expression analysis specifically on these two groups. It resulted that although the GEX profile of CAIT and MAIT cells is very similar, CAIT cells express higher levels of natural-killer associated genes, such as *KLRD1, GZMH, FGFBP2* and *CX3CR1*, while they downregulate *IL7R* (figure 5F and online supplemental table S7E).^34 35^ Additionally, CAIT cells expressed *CD69*, *IFNg* and *TNF* too (online supplemental table S7E).

Individual CAIT and MAIT cells were additionally found in the CD4^+^ memory clusters, confirming our observations at bulk TCR level, that CAIT clonotypes occur in both fractions with an enrichment in CD8^+^ cells (figure 4A, B)

### Variable frequency of CAIT clonotypes among TRAV12-1+ cells

Next, we wanted to evaluate whether it is possible to efficiently capture and characterise CAIT clonotypes by flow cytometry. We analysed the peripheral blood of 39 patients with CD, 20 patients with UC and 18 healthy controls, as well as gut biopsies from 14 patients with CD, 7 patients with UC and 19 patients with CRC (online supplemental tables S8 and S9). By gating on CD3^+^TRAV12-1^+^CD161^+^IL18R^+^ cells (gating strategy in online supplemental figure 4A), we found a significant enrichment of this population in the blood of CD, and surprisingly also in patients with UC, as compared with healthy controls (online supplemental figure 4B). Among these cells, the CD8^+^ fraction (CD8^+^TRAV12-1^+^CD161^+^IL18R^+^) was clearly enriched in patients with CD as compared with healthy donors (online supplemental figure 4C). Contrarily, as shown before by other studies,^17^ MAIT cells (CD3^+^TRAV1-2^+^CD161^+^IL18R^+^) were decreased in the blood of patients with IBD both in the total CD3^+^ and the CD3^+^CD8^+^ compartment (online supplemental figure 4D, E). However, analyses of gut biopsies did not fully recapitulate these findings. Although the ‘CAIT-like’ population (CD3^+^TRAV12-1^+^CD161^+^IL18R^+^) had in general a higher frequency in the intestinal tissue (0%–4% of CD3^+^cells) as compared with blood (0%–1.8% of CD3^+^cells), we observed only a trend, but no significant enrichment of this population in CD (online supplemental figure 4F-H), contrarily to MAIT cells (online supplemental figure 4I, J).

**Figure 6:**
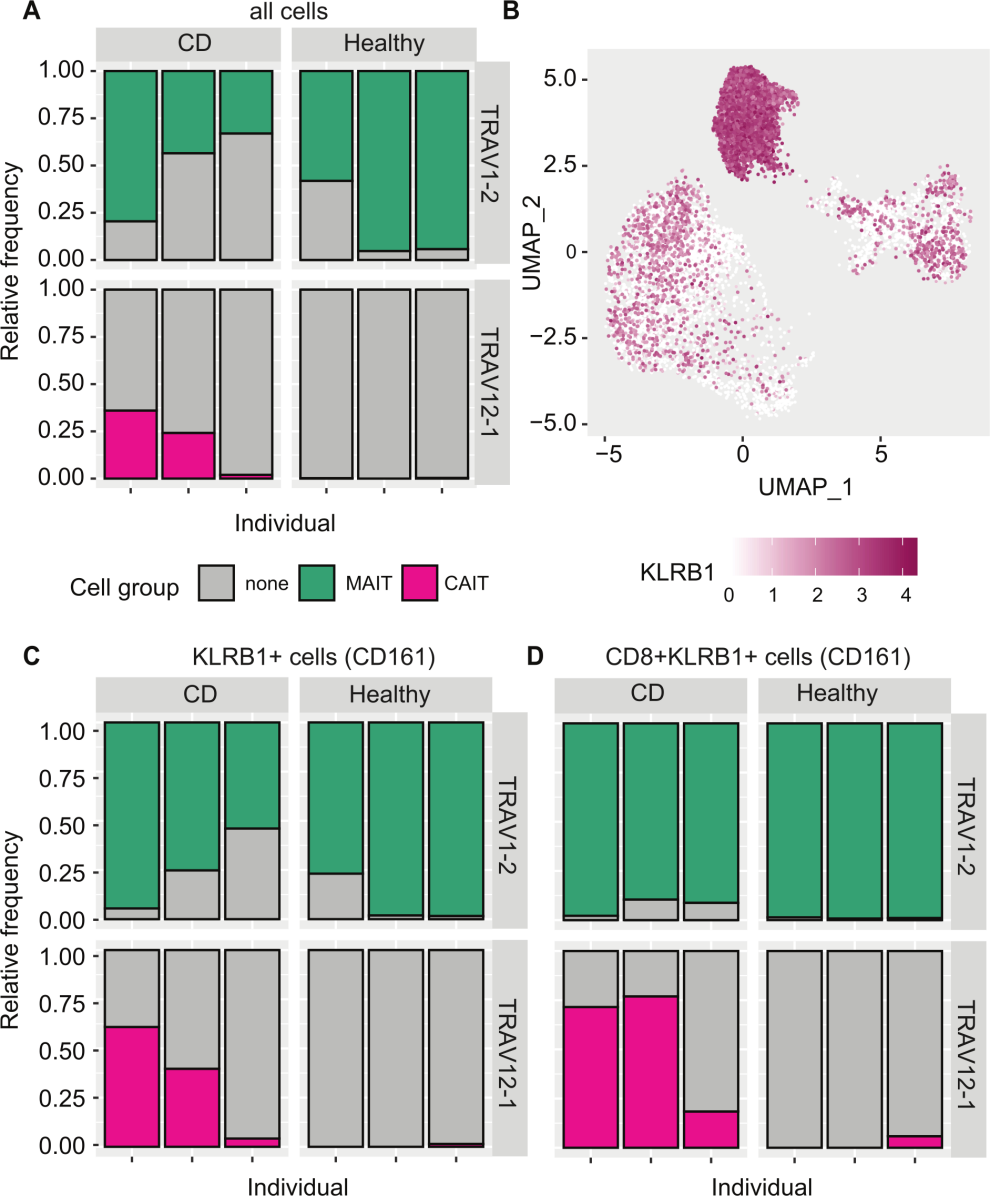
Mucosal associated invariant T (MAIT) and Crohn-associated invariant T (CAIT) cell distribution in TRAV1−2^+^ and TRAV12−1^+^ cells the single-cell dataset. TRAV1-2^+^ and TRAV12-1^+^ cells were selected from the single-cell dataset in figure 5. (A) Distribution of MAIT and CAIT cells among the three patients with CD and three healthy controls samples for TRAV1-2^+^ and TRAV12-1^+^ cells. (B) Distribution of the expression levels of KLRB1. The expression is higher in the same cell groups where MAIT and CAIT cells mostly localise. (C) Distribution of MAIT and CAIT cells in the cell subset expressing KLRB1, encoding for the CD161 protein. (D) Distribution of MAIT and CAIT cells in the CD8^+^ cell subset expressing KLRB1.

Specific clonotypes cannot be captured by flow cytometry if the HLA epitope is unknown and thus the accuracy of their detection is limited to the TCR V region, for which specific antibodies are commercially available. Therefore, we used our single-cell data to assess the frequency of CAIT clonotypes among clonotypes expressing a TCR using the *TRAV12-1* gene. We also compared it with the frequency of MAIT clonotypes among TRAV1-2^+^ cells. MAIT TCRs accounted for 33%**–**80% of the analysed TRAV1-2^+^ cells in patients with CD and 58%**–**95% in healthy controls. In contrast, the percentage of CAIT clonotypes among TRAV12-1^+^ cells was highly variable, ranging from 1.9% to 36% in patients with CD, and 0% to 0.3% in healthy controls (figure 6A). The frequency of CAIT cells was increased when focussing on cells expressing *KLRB1* (figure 6B), encoding for the CD161 protein. In this subset, CAIT clonotypes were 6.0%**–**70% of TRAV12-1^+^ cells in patients with CD and 0%**–**2% in healthy controls, while the proportion of MAIT clonotypes among TRAV1-2^+^ was 64%**–**96% in patients with CD and 82%**–**99% in controls (figure 6C). Maximum frequencies were reached in the TRAV12-1^+^CD161^+^CD8^+^ cells where CAIT cells ranged from 18% to 77% in patients with CD and 0% to 5.8% in healthy controls (figure 6D).

These GEX data suggest that while MAIT clonotypes are strongly represented in a flow cytometry analysis of TRAV1-2^+^CD161^+^ T cells, CAIT clonotypes have high frequency in some individuals but low frequency in others. Therefore, although GEX levels do not directly translate into protein expression levels, it is reasonable to hypothesise that TRAV12-1^+^CD161^+^ cells analysed via flow cytometry include CAIT clonotypes but that the high variability between individuals limits the use of flow cytometry for their detection.

## Discussion

By means of high-throughput TCR profiling, we here identified a specific group of T cells, defined by a semi-invariant TCR alpha motif present in both blood and intestinal tissue. CAIT clonotypes enrichment in CD was significant in blood and mesenteric lymph nodes while only a trend was observed in intestinal tissue. Potentially, these cells may be activated into the gut and then enter the peripheral circulation. Their accumulation in peripheral blood may also be a sign of systemic disease. We did not observe correlations between occurrence of the CAIT TCR alpha motif and clinical parameters or other confounding factors.

We observed CAIT cells to be present in both CD4^+^ and CD8^+^ fractions, but further characterisation through single-cell GEX of presorted blood memory CD4^+^ and CD8^+^ T cell fractions showed higher enrichment in the CD8^+^ fraction. Single-cell data also revealed the alpha/beta TCR pairs of CAIT cells. Pairing with diverse beta chains was observed, with a preferential usage of the *TRBV7-9* gene.

CAIT cells were mostly found in CD8^+^ effector memory clusters, with a compatible phenotype to unconventional T cells as MAIT and NKT cells. Innate-like cell markers such as *KLRB1* (CD161), *SLC4A10, CEBPD, IL18R* and *KLRD1* (CD94) were indeed highly expressed by CAIT cells. In contrast to MAIT cells, that are known to be decreased in the blood of patient with CD, CAIT clonotypes were significantly increased in our patients’ blood, suggesting a different role of CAIT cells in CD. Despite the high abundance of CAIT clonotypes in certain subjects, the proportion of these cells among individuals varies greatly. Therefore, T cell populations capturable by flow cytometry, based on our current analysis, that is, TRAV12-1^+^CD161^+^, are not specific enough to further dissect the immunophenotype and functional profile of CAIT clonotypes. The observed differences of flow-cytometric detection in blood versus intestinal tissue may also relate to a different cell composition of the TRAV12-1^+^CD161^+^ compartment, which needs to be investigated in future studies. Also, disease activity and disease location may have an impact on the observed results, as mostly colonic biopsies and patients in disease remission could be obtained for flow cytometric analysis. Thus, although TCR analyses highlighted the presence of CAIT clonotypes in the gut, further analyses stratifying patients not only by disease condition but also by frequency of CAIT cells among CD3^+^, will be necessary to clarify the role of these clonotypes in the intestinal tissue and in IBD.

Recently, Almeida *et al* published a study characterising a subset of CD1d-reactive NKT type II cells.^19^ Apart for MAIT cells, so far only the CD1d-reactive iNKT cells are known to have a restricted TCR alpha repertoire.^15^ NKT type II cells are described to have an oligoclonal repertoire.^15^ The cells described by Almeida showed indeed a polyclonal TCR repertoire, but with preferential usage of the *TRAV12-1*, *TRAJ6* gene combination, the same observed in CAIT clonotypes. The main GEX marker of NKT type II cells in humans is CD161, thus fitting the phenotype of CAIT cells.^15 18^ Moreover, two of the TCR alpha clonotypes therein described fit the CAIT CDR3 motif (figure 1B).^19 36^ Given these results, we hypothesise that CAIT cells may be reactive to CD1d and thus be a semi-invariant subgroup of the NKT type II family. Indeed, identification of HLA and antigen specificity is an essential step in T cell characterisation. We did not observe a correlation with a specific classical MHC allele in the patients with high abundance of CAIT cells, suggesting that classical MHC-restriction is indeed not driving the expansion of these cells. This hypothesis was further supported by the fact that CAIT cells were found in both the CD4^+^ and the CD8^+^ compartments.

The cells identified by Almeida *et al* are reactive to small lipids produced by the microbiome as well as to PentamethylBenzofuranSulfonates (PBFs). Therefore, CAIT cells may be potentially reactive towards microbiome metabolites or small lipids, which were recently shown to modulate NKT cell responses in the gut.^37^

The study herewith described also has limitations that we want to highlight here. In our study, we were able to control for only some confounding factors such as age, sex, disease activity, smoking behaviour and anti-TNF treatment, while other factors such as medication and disease history may impact on the observed cell population. Moreover, CD is a very heterogeneous disease with high interindividual variability, demanding larger sample sizes than examined here to define larger subgroups for stratified analyses. Additionally, control intestinal samples were available only for non-inflammatory disease controls (CRC) but not healthy individuals, which is another limitation of our study. Finally, technical limitations and interindividual variability do not allow for efficiently capture CAIT cells via flow cytometry. Thus, further studies, including longitudinal sampling strategies, will be necessary to better assess the effect that medications, as well as the transition from active disease to disease remission, may have on CAIT cells.

In summary, we describe a new semi-invariant subgroup of unconventional T cells to be specifically enriched in the blood of patients with CD. Evidences suggest these cells to be NKT type II cells. The consequences of this enrichment remain to be clarified, and CAIT cells immunophenotype and antigen reactivity need to be further elucidated in future studies. Finally, given the semi-invariant nature of CAIT cells, similarly to MAIT and iNKT cells, they are likely to be public in humans at population level, thus constituting an interesting target of study in both health and disease.

## Materials and methods

### Study design: summary

Because dysregulated T cell reactions against intestinal antigens are considered causal or driving factors for CD, we aimed at identifying potentially existing disease-associated specific T cell clonotypes through the analysis of the TCR repertoire. Identification of disease-associated TCRs may shed light on specific dysregulated T cell responses and may take us a step closer to the identification of potential antigenic triggers of the disease. In our initial analysis, blood samples of patients with CD were selected from the available baseline samples of a prospective registry of patients with CD undergoing treatment with biologics which was established by the German ‘Competence Network IBD’, Kiel, Germany in 2008. Additional goals included investigation of correlation between the identified disease-associated TCRs^20 38–42^ and genotypes^43^ and clinical phenotypes of the patients. Therefore, patients included in our initial analysis were characterised by active disease status^44–47^ and were known to be responding or non-responding to anti-TNF therapeutic treatment after 6 months from baseline sampling. Age-matched and sex-matched healthy controls were selected from a local registry of blood donors. UC samples were selected from a patient cohort collected at the local hospital to verify the disease specificity of the identified CD-associated TCRs. Finally, 109 patients with CD, 36 patients with UC and 99 healthy controls were included in the analysis. Samples excluded because of failure in library preparation, sequencing or because not passing quality filtering were not included in the manuscript. A second sample collection was analysed to validate the findings in the first collection and to investigate presence and abundance of the identified Crohn-associated clonotypes in the intestinal tissue. Surgical specimens were collected from patients undergoing bowel resection (N*=*37). Patients with CRC were included as disease controls. It is important to notice that the used intestinal tissue from patients with CRC was not tumour tissue, but adjacent, macroscopically healthy, tissue. T cell subpopulations were sorted from blood and gut of an additional subgroup of patients (N*=*21) to investigate the cellular population of origin of the identified Crohn associate clonotypes. To investigate in detail the phenotype of the disease-associated cells as well as the TCR alpha/beta pairing, the T cell populations most enriched for these cells, namely CD4^+^ and CD8^+^ memory cells, were sorted from freshly collected blood of three recontacted patients with CD of the surgery sample collection and underwent single-cell TCR-seq and RNA-seq.^27 48 49^

Detailed methodologies are described in the online supplemental materials and methods.

## Data Availability

Data are available upon reasonable request. Data may be obtained from a third party and are not publicly available. Raw sequencing data of bulk TCR repertoire profiling are available on the ENA database with study accession number PRJEB50045. Single-cell gene expression and TCR processed data are available on the FastGenomics database (Seurat_objects_Rosati_TCR_IBD). Raw single cell data (fastq files) are available from the corresponding author upon request. HLA genetic data are available from the corresponding author upon reasonable request. Metadata from the whole blood TCR collection are available through the Popgen biobank.
