# Supplementary material for: A novel unconventional T cell population enriched in Crohn’s disease
Source: Gut. 2022 Mar 9;71(11):2194–204. doi: 10.1136/gutjnl-2021-325373 (PMC9554086; doi:10.1136/gutjnl-2021-325373)
Supplement: Supplementary data [file gutjnl-2021-325373supp001.pdf]

*Rosati et al. - A novel unconventional T cell population enriched in Crohn's disease*

# SUPPLEMENTARY MATERIALS

## Supplementary Material and Methods

**Supplementary Table S1: summary of performed analyses and used sample collections**

| Dataset | Sample type | Analysis | Disease group | Number of individuals |
|---|---|---|---|---|
| 1 whole blood | PAXgene tubes | Bulk TCR repertoire + HLA imputation | CD | 109 |
| | | | UC | 36 |
| | | | Healthy | 99 |
| 2.1 surgery | PBMCs + Gut biopses + Mesenteric lymph nodes | Bulk TCR repertoire | CD | 11 |
| | | | UC | 13 |
| | | | CRC | 13 |
| 2.2 surgery sorted | Sorted T cells from PBMCs + Sorted T cells from gut | Bulk TCR repertoire | CD | 7 |
| | | | UC | 5 |
| | | | CRC | 9 |
| 3 single-cell | Sorted T cells from PBMCs | Single-cell RNA and TCR | CD | 3 |
| | | | Healthy | 3 |

### Whole blood sample collection

Phenotypic details for this collection are described in **Supplementary Table S2**.

*CD patient collection:* BioCrohn is a non-interventional prospective registry of Crohn's disease patients undergoing biologics treatment which was established by the German "Competence Network IBD", Kiel, Germany in 2008. Samples included in this study were collected prior to the start of anti-TNF therapy (Infliximab).

To evaluate disease activity, we use the Harvey-Bradshaw Index (HBI). We employ this measure to distinguish between inactive (0≤HBI≤4: remission) and active state of the disease (5≤HBI≤7: mild activity, 8≤HBI≤16: moderate activity, 17≤HBI≤∞: severe activity)(*1*), as described from the Canadian society of intestinal research. In total, whole blood samples of 109 CD patients were available for analysis. All patients had active disease at sampling time. Response to anti-TNF therapy was defined as a steroid-independent change from active disease (HBI > 4 at baseline) to an inactive state of the disease (HBI < 4 at 6 months after start of therapy). Accordingly, we defined non-response to anti-TNF therapy as the disease being consistently active at both time points under investigation (HBI > 4 at baseline and 6 months).

*UC patient collection:* blood samples from UC patients of the "whole blood collection" were recruited as part of a larger panel of patients with IBD, recruited in the Comprehensive Centre for Inflammation Medicine (CCIM), at the University Hospital Schleswig-Holstein, Campus Kiel (Germany). Samples

for the present study were randomly selected among the available samples stored in the PopGen biobank. In total, whole blood samples of 36 UC patients were available.

*Healthy control collection:* blood samples were obtained from the PopGen control sample, consisting of a community-based sample from the Kiel area (Northern Germany) and of blood donors from the University Hospital Schleswig-Holstein, Campus Kiel(*2*). In total, 1316 individuals were recruited at baseline (between 2005 and 2007) and were re-examined at the second examination cycle; between 2010 and 2012; n=952)(*3, 4*). Participants filled-in questionnaires on health and nutrition, received a basic clinical examination and provided blood samples for research. For the present analyses, individuals with major diseases such as cancer, neurological and inflammatory diseases were excluded. In total, 99 whole blood samples were available. These samples were matched to the CD patients regarding age and sex.

2.5 ml of blood were drawn and stored in PAXgene tubes (Qiagen), following the manufacturer's instructions. Samples were stored at -80°C. Total RNA was isolated using the PAXgene blood miRNA kit (Qiagen) with automatic isolation using the Qiacube machine. 1,200 ng of total RNA were used for TCR library preparation.

*Rosati et al. - A novel unconventional T cell population enriched in Crohn's disease*

***Supplementary Table S2: Whole blood collection phenotype information.*** Information about disease phenotype was provided via the Montreal classification. Information on the disease activity was available through the Harvey-Bradshaw index (HBI) for CD patients and though the colitis activity index (CAI) and partial Mayo score for UC patients. Information about steroid therapy at sampling time was also provided.

| | Sample group | | |
|---|---|---|---|
| | **CD** | **UC** | **Healthy** |
| **Basic phenotype** | | | |
| Samples | 109 | 36 | 99 |
| Gender, Male/Female (%) | 43/65 (40/60%) | 21/15 (58/42%) | 41/58 (41/59%) |
| Age, mean±std | 40±12 | 37±13 | 48±13 |
| **Montreal classification** | | | |
| *Age at diagnosis in years* | | | |
| A1, <16) (%) | 7 (6%) | – | – |
| A2, 17-40 (%) | 74 (70%) | – | – |
| A3, >40 (%) | 25 (24%) | – | – |
| *Behaviour* | | | |
| B1, inflammatory (%) | 51 (47%) | – | – |
| B2, stricturing (%) | 21 (19%) | – | – |
| B3, penetrating (%) | 36 (34%) | – | – |
| *Location* | | | |
| L1, ileal (%) | 29 (27%) | – | – |
| L2, colonic (%) | 23 (21%) | – | – |
| L3, ileocolonic (%) | 56 (52%) | – | – |
| *Disease extent* | | | |
| E1, proctitis (%) | – | 3 (3%) | – |
| E2, left-sided UC (%) | – | 16 (47%) | – |
| E3, extensive UC (%) | – | 15 (44%) | – |
| Info not available (NA) | 1 | 2 | – |
| **Disease activity** | | | |
| HBI, mean±std | 11±5 | – | – |
| Mild activity, 5≤HBI≤7 (%) | 37 (34%) | – | – |
| Moderate activity, 8≤HBI≤16 (%) | 57 (53%) | – | – |
| Severe activity, 17≤HBI≤∞ (%) | 14 (13%) | – | – |
| Partial Mayo score, mean±std | – | 3.3±2 | – |
| CAI, mean±std | – | 6.7±4.3 | – |
| Info not available (NA) | 1 | 0 | – |
| **Medications** | | | |
| Steroids, yes/no (%) | 7/102 (6/94%) | 14/22 (39/61%) | – |
| Treatment naïve yes/no/NA (%) | 77/14/18 (70/13/17%) | 0/0/36 (0/0/100%) | – |
| Responders to anti-TNF yes/no/NA (%) | 46/60/3 (42/55/3%) | – | – |

## Surgery sample collection

Samples were collected in collaboration with the *Clinic for General and Thoracic Surgery* of the University Hospital Schleswig-Holstein, Kiel, Germany. The study was approved by the ethics committee of the medical faculty of the Christian-Albrecht University Kiel, Kiel, Germany (ethics vote: D553/16). A total of 37 patients were recruited (11 CD, 13 UC, 13 CRC). It is important to notice that for CRC tissue samples, macroscopically normal, non-tumour tissue was utilized. Sample details are provided in **Supplementary Table S5**. Information on disease activity scores or treatment with biologics prior to operation was unfortunately not available. However, we did not see a correlation with these parameters in the whole blood collection.

Sample collection included **(1)** one 9 ml whole blood EDTA tube from which PBMCs were isolated by density gradient centrifugation (Ficoll, GE Healthcare) and **(2)** resected intestinal mucosa. Tissues were cut into pieces of approximately 30–50 mg, snap-frozen and stored at -80°C. Details on the number of collected samples and intestinal resection location for each patient are available in **Supplementary Table 3**, together with patients' basic phenotypic information.

Total RNA was isolated using the RNeasy mini kit from Qiagen following the manufacturer's instructions. Up to 3,200 ng of total RNA were used for TCR library preparation.

***Supplementary Table S5: Surgery collection phenotype information***. Information about the numbers of collected samples for each tissue is provided together with information about intestinal location of the sample and about therapy with steroids or immunosuppressants at time of surgery. NA: no information available.

| | CD | UC | CRC |
|---|---|---|---|
| **Basic phenotype** | | | |
| Samples | 11 | 13 | 13 |
| Gender, Male/Female (%) | 4/7 (36/64%) | 7/6 (54/46%) | 8/5 (62/38%) |
| Age, mean±std | 40±15 | 52±16 | 69±12 |
| **Sample types** | | | |
| Intestinal samples | 11 | 13 | 9 |
| Mesenteric lymph nodes | 4 | 5 | 0 |
| PBMCs | 11 | 12 | 12 |
| Intestine + PBMCs datasets | 11 | 12 | 8 |
| **Resected tissue location** | | | |
| *Large intestine* | 4 | 13 | 13 |
| *Small intestine* | 7 | 0 | 0 |
| **Medications** | | | |
| Steroids, yes/no/NA | 4/5/2 | 7/6/0 | 0/0/13 |
| Immunosuppressants, yes/no/NA | 5/5/1 | 2/10/1 | 0/0/13 |

## Surgery sample collection: sorted T cell populations

Additional 21 patients (9 CRC, 7 CD, 5 UC) were recruited for the surgery collection (**Supplementary Table S6**). PBMCs were isolated from peripheral EDTA blood samples by density gradient centrifugation (Ficoll, GE Healthcare).

To generate single cell suspensions from intestinal mucosa, resected tissue was washed twice in sterile PBS. Mucosa was separated from the muscle layer and fat and cut into small pieces. Mucosa pieces were digested in digestion buffer containing 1xHBSS+calcium+magnesium buffer (Gibco) supplemented with 2% human AB serum (Sigma Aldrich), 100 IU/ml penicillin, 100 µg/ml streptomycin, 0.25 µg/ml amphotericin B (Antibiotic Antimycotic Solution, Sigma Aldrich), 1mg/ml Collagenase NB (Nordmark Biochemicals) and 200µg/ml DNAse I (AppliChem) for 75min at 37°C under continuous rotation at a ratio 3ml/g of tissue. After digestion, the supernatant was filtered using a 100µm filter, followed by a 70µm filter. Remaining tissue pieces were squeezed through a sterile sieve, filtered and combined with the supernatant. Cells were pelleted by centrifugation for 10min at 500xg, followed by a red blood cell lysis (Red Blood Cell Lysis Solution, Miltenyi Biotec).

***Supplementary Table S6: Sorted surgery collection phenotype information.*** Information about the numbers of collected samples for each tissue is provided together with information about intestinal location of the sample.

| | Sample group | | |
|---|---|---|---|
| | **CD** | **UC** | **CRC** |
| **Basic phenotype** | | | |
| Samples | 7 | 5 | 9 |
| Gender, M/F (%) | 3/4 (43/57%) | 1/4 (25/75%) | 8/1 (89/11%) |
| Age, mean±std | 39±11 | 42±17 | 69±17 |
| **Sample types** | | | |
| Intestine + PBMCs datasets | 6 | 5 | 9 |
| **Resected tissue location** | | | |
| *Large intestine* | 2 | 5 | 8 |
| *Small intestine* | 5 | 0 | 1 |

To isolate different T cell populations, $CD4^+$ T cells were isolated by MACS (CD4 MicroBeads, Miltenyi Biotec) and $CD4^+$ and $CD4^-$ cells were stained with the following antibodies according to manufacturers' protocols:

<u>$CD4^+$ cells</u>: CD3-AlexaFluor700 (OKT-3), CD4-BV421 (OKT-4) (both Biolegend), CD45RO-FITC (UCHL1), CCR7-PE-Vio770 (REA108), CD25-PE (REA613), CD127-APC (REA614), CD8-PerCP (BW135/80), CD14-PerCP (Tük4), CD20-PerCP (Tük4) (all MiltenyiBiotec).

<u>$CD4^-$ cells:</u> CD3-PE (BW264/56), CD8-APC (BW135/80), CD14-PerCP (Tük4), CD20-PerCP (Tük4) (all MiltenyiBiotec). Propidiumiodide was used to exclude dead cells. Cells were sorted on a BD FACS ARIA into the following populations:

$CD4^+$ T memory ($CD3^+CD4^+CD45RO^+CD127^+CD25^-CD8^-CD14^-CD20^-$)

$CD4^+$ T naïve ($CD3^+CD4^+CD45RO^-CCR7^+CD127^+CD25^-CD8^-CD14^-CD20^-$)

CD4$^+$ Treg (CD3$^+$CD4$^+$CD45RO$^+$CD127$^-$CD25$^+$CD8$^-$CD14$^-$CD20$^-$)

CD8$^+$ T cells (CD3$^+$CD8$^+$CD14$^-$CD20$^-$)

Total RNA was isolated using Trizol and was used for TCR library preparation.

## Genotyping & HLA imputation

DNA extraction was performed by the DNA laboratory of the Institute of Clinical Molecular Biology (Christian-Albrechts-University of Kiel, Germany) using a Chemagic 360 from PerkinElmer (Waltham, Massachusetts, U.S.) with the low volume kit cmg 1491 and the buffy coat kit cmg-714 (Chemagen, Baesweiler, Germany) according to the manufacturer's protocol. Genotyping was conducted by the Institute of Clinical Molecular Biology's DNA Laboratory and Genotyping Core Facilities, employing Illumina's (Illumina Inc., San Diego, U.S.) Global Screening Array-24 Multi Disease (GSA) Version 1.0 A1 (whole blood collection: CD cases) or at the at the Regeneron Genetics Center using the same array (whole blood collection: healthy controls). The GSA chip highly covers the HLA region, with 9,876 variants on the extended HLA on chromosome 6 from 25Mb to 34Mb.Due to the small sample size numbers, we did not perform a special QC on the variants in the HLA region. The overall genotyping rate in both studies was > 98%. Based on the SNP data we excluded 2 individuals not matching to a European ancestry, none of the individuals in the case or control cohort were related with an identity-by-descent > 0.185, all recorded phenotypic and genotypic gender information matched. HLA genotypes were imputed at all classical HLA class I (HLA-*A, -B, -C)* and HLA class II (HLA-*DRB1/3/4/5*, *-DQA1*, *-DQB1*, *DPA1*, *-DPB1*) loci at full context 4-digit resolution using our previously published in-house trans-ethnic HLA imputation reference(*5*) based on the Illumina Immunochip, which we retrained using only the positions present on both the Illumina Immunochip and GSA Version 2.0 B1 which is highly similar to the content of the GSA Version 1.0 A1, and HIBAG(*6*) (https://hibag.s3.amazonaws.com/download/IKMB_HMG/index.html). Overall, 98% of all SNPs per locus out of 4,574 SNPs present in the HLA reference dataset overlapped between the reference and the GSA data.

SNPs not matching between the reference and the respective imputed dataset (based on their alleles) were excluded, ATCG variants with a MAF frequency of >40% or those not matching the reference (+-strand annotation assumed for both) were deleted from the dataset. Post imputation data were not cleaned according to any imputation threshold. However, measures such as the marginal probability of alleles(*7*) and similarity of alleles based on the SNP overlap between classifiers(*5*) were taken into consideration for the interpretation of results.

## TCR library preparation and sequencing

Starting from total RNA, molecular-barcoded TCR cDNA libraries were prepared as previously described(*8*), with minor modifications for both TCRα and TCRβ chains. Briefly, cDNA synthesis was

performed using SMARTScribe reverse transcriptase (Clontech, Takara) using primers for the TCRα and TCRβ constant region. A unique molecular identifier (UMI), and a sample barcode of 6 nucleotides, were introduced via template-switching. cDNA synthesis was carried out for 60 minutes at 42°C. cDNA was then treated with Uracil DNA-Glycosylase (UDG, from New England Biolabs) and incubated for 30 min at 37°C. Samples were subsequently purified with the QIAquick PCR purification kit (Qiagen) and eluted in 50 or 100 µl deionized water. Purified cDNA was then amplified with 2 consecutive PCRs, respectively 18 and 12 cycles, with purification after each PCR using MagSi-NGSprep Plus (MagnaMedics). Illumina compatible adapters and sample-specific barcodes were added during the second PCR. Quality and concentration of the libraries were measured with TapeStation D1000 (Agilent) and Qubit (Thermofisher). Libraries were pooled using 5 ng per library and sequenced on Illumina HiSeq2500 with a single-index Rapid Run of 2×100 bp (whole blood) or on Illumina NovaSeq 6000 SP 2×150 bp flow cell (surgery). Custom sequencing primers were added to the Illumina primers. For the sorted surgery collection samples, libraries were prepared using human TCR profiling kit (MiLaboratory), according to the manufacturers' protocol. Libraries were sequenced on Illumina MiSeq 2×150 bp or NovaSeq 6000 SP 2×150 bp.

## TCR data pre-processing

PCR and sequencing error correction were performed through identification and selection of unique molecular identifiers using the software MiGEC(*9*), version 1.2.6. Filtered sequences were aligned on a TCR gene reference, clonotypes were identified, grouped and CDR3 sequence was identified using the software MiXCR(*10*), version 2.1.1 (first sample collection: blood collection) or version 3.0.14 (second collection: surgery collection). Clonotype tables containing clonotype counts, frequencies, CDR3 nucleotide and amino acid sequences and V(D)J genes were obtained and used for further analysis.

Summary data on all analysed samples, including the total number of identified clonotypes per sample, are available in **Supplementary Table S3A**. A total of 444 samples were analysed for bulk TCR repertoire.

## TCR data analysis

For statistical analysis, the Wilcoxon-Mann-Whitney U test was used unless otherwise indicated. Samples with less than 200 identified unique clonotypes were excluded from the analysis. TCRs found at a count inferior to 2 (singletons) or encoding for out of frame sequences or TCRs containing stop codons, were also excluded from further analyses.

**Proportion analysis of TCR groups (CDR3length_Vgene_Jgene)**: For each disease group (CD, UC, healthy), the total number of TCR sequences being part of each TCR group (CDR3length_Vgene_Jgene) was calculated and divided by the number of total TCR sequences present

in each disease group in order to normalize for differences in TCR number among disease groups. The proportion of TCRs of each TCR group was then compared between disease groups, either CD against UC, or against healthy controls, for both TCR alpha and TCR beta.

**Fisher test on identified TCR groups of interest:** using specific CDR3 and VJ gene combinations identified through the analysis of repertoire proportions (12aa_TRAV1-2_TRAJ33, 15aa_TRAV12-1_TRAJ6), we performed one-sided Fisher's exact test on the single TCRs of these groups to identify TCR alpha clonotypes respectively decreased (12aa_TRAV1-2_TRAJ33) or increased (15aa_TRAV12-1_TRAJ6) in CD patients versus healthy individuals, from the whole blood and surgery collections. We applied multiple testing correction through the Benjamini-Hochberg (BH) method(*11*), in order to control expected proportion of false discoveries amongst the rejected hypotheses (false discovery rate, FDR).

**Analysis on CAIT sequences:** TCR sequences from the identified group of CDR3length = 15 amino acids, V gene = TRAV12-1 and J gene = TRAJ6 that were found to be more abundant in CD patients as compared to controls in the whole blood sample collection were used for the logo plot which defined the CAIT clonotype motif CVV**A*GGSYIPTF. All sequences of the selected TCR group, carrying the CAIT motif were selected for further analysis. For each sample, the number of CAIT sequences present in the sample as well as the sum of relative abundances (cumulative abundance) of CAIT sequences were calculated.

For the network plots, CAIT sequences of each sample were used. Their relative abundance was divided in discrete groups as indicated in the figure legends and defined the size of the vertexes of the network. Each sample was represented by nodes connected by edges. Separated clusters represent distinct samples/individuals. Figure 2E was generate using the R igraph package(*12*).

**Association of CAIT clonotypes with phenotype and genotype traits:** A generalized linear model was fitted using as dependent variable either the cumulative abundance or the number of CAIT clonotypes in each sample and as independent variables the clinical parameters which were, for CD patients: age (as from the Montreal classification), sex, smoking behaviour, disease location (as from the Montreal classification), disease activity (based on HBI), prediction response to anti-TNF therapy, and for healthy controls: age, sex and smoking behaviour. A similar model was fitted for each analysed HLA allele using as independent covariates the dosage of each allele, where 0 indicated absence, 1 heterozygosity and 2 homozygosity for the analysed allele. The used formulas for each model are described in **Supplementary Table S4**.

### TCR sequence and structural analysis

The protein structural model of the exemplary CAIT TCR (TRAV12-1- CVVNLASGGSYIPTF - TRAJ6- / TRBV7-9 – CASSTRELANTIYF - TRBJ1-3) was constructed using the TCRmodel webserver(*13*) and visualized using PyMOL.

## Single-cell analysis

Blood was drawn from three CD patients, 2 females and 1 male of age 29-, 49- and 36-year-old respectively, who were shown to have high abundance of CAIT TCRs during bulk TCR analysis, as well as from three age and sex matched healthy controls. PBMCs were isolated by gradient centrifugation from EDTA blood tubes. CD3+ cells were enriched by MACS (Miltenyi Biotec).
For single-cell analysis, $CD3^+$ (VioBle) $CD45RO^+$ (PeVio770) memory T cells were sorted in $CD8^+$ (VioGreen) and $CD4^+$ (APCVio770) subpopulations. Cells were removed from the sorting chamber into pre-coated low-bind collection tubes and centrifuged for 5 min at 400x g, 4°C. Cells were re-suspended in PBS plus 0,04% BSA to a concentration of 1000 cells/ul.

Single-cell suspensions were loaded on a Chromium Chip G (10x Genomics) according to manufacturer's instructions for processing with the Chromium Next GEM Single Cell 5' Library and Gel Bead Kit v1.1. Depending on the number of cells available for each patient, a maximum of 20,000 cells were loaded for each reaction. TCR single-cell libraries were subsequently prepared from the same cells with the Chromium Single Cell V(D)J Enrichment Kit, Human T Cell. Libraries were sequenced on the Illumina NovaSeq 6000 machine with 2×100 bp for gene expression, aiming for 50,000 reads per cell and 2×150 bp and 5000 reads per cell for TCR libraries.

Single-cell T cell receptor repertoire clonotype tables were generated using the VDJ command of the Cellranger software, version 3.1.0 from 10xGenomics and using the VDJ reference version 2.0.0. Clonotype tables were filtered to include only cells which passed quality filtering in the gene expression analysis. In addition, clonotypes were stringently filtered for possible doublets by removing clonotypes (i) found in 1 cell only and containing more than 1 TCR alpha and 1 TCR beta (ii) containing more than 1 TCR alpha and no TCR beta sequence (iii) containing more than 1 TCR beta and no TCR alpha sequence (iv) containing more than 2 TCR alpha or more than 2 TCR beta sequences.

Gene expression matrices were generated through the COUNT command of Cellranger v3.1.0. from 10xGenomics and using the reference GRCh38 version 3.0. Data were analyzed using the Seurat v3.2.3 R package(*14*). Cells with more than 400 but less than 3500 detected RNA features per cell and less than 8% mitochondrial RNA were retained for further analysis. Only genes present in at least 1% of the cells were considered. Also, only cells with a detected TCR were used. TCR VDJ genes were removed from gene expression counts to allow for an unbiased analysis. Data from multiple individuals were merged and batch effect correction by experimental day and sequencing run was performed using the Harmony package(*15*). 20 dimensions were used for performing principal component analysis (PCA) and uniform manifold approximation and projection (UMAP) in 3 dimensions. Clusters were identified using 0.4 resolution. Positive and negative marker genes were identified using Seurat FindMarkers function and the MAST method(*16*), considering only genes found in at least 25% of cells of each cluster.

MAIT cells were identified by their TCR alpha sequences (12 aa CDR3, TRAV1-2, TRAJ33/12/20), as well as iNKT cells (15aa CDR3, TRAV10, TRAJ18), while CAIT cells were defined from TCR alpha of 15aa, TRAV12-1, TRAJ6 and CDR3 motif CVV**A*GGSYIPTF. Proportion of CAIT cells carrying certain TRBV genes was calculated based on the number of unique CAIT clonotypes identified, independently from the number of cells carrying each clonotypes and originated by the same original clonotype by clonal expansion.

## Immunophenotyping by flow cytometry

Human mononuclear cells were isolated from whole blood by standard density gradient centrifugation (Biocoll, Biochrom, Berlin Germany). Intestinal tissue was incubated in HBSS media with 20uL/mL DNAse, 1mg/mL collagenase, 1% penicillin/streptomycin and 0,2% human AB-serum for 30 min in a shaking incubator at 37°C. Biopsies were then dissociated by mechanical pressure and strained through a 70µm filter. Cells were washed with RPMI medium. Cells from blood and tissue freshly after isolation were stained on the surface using an antibody cocktail. All flow cytometry plots are gated on CD14-, CD20-, CD3+ lymphocytes after dead cell and doublet exclusion.

## Whole blood and intestinal tissue biopsy sample collection for flow cytometry immunophenotyping

*Crohn's disease and Ulcerative colitis patients:* Samples were collected in collaboration with the *Comprehensive Center for Inflammation Medicine (CCIM)* of the University Hospital Schleswig-Holstein, Kiel, Germany. A total of 25 CD patients and 13 UC patients were recruited (including 2 from the '*Whole blood collection used for TCR repertoire sequencing').* Patient information is provided in **Supplementary Table S8**. An additional collection of patients with matching peripheral blood and intestinal biopsy samples was collected, 14 CD patients and 6 UC patients (**Supplementary Table S9**). Therefore, in total we analysed blood samples for 39 CD patients (25 + 14) and 19 UC patients (13 + 6).

*Colon cancer patients as disease controls:* Fifteen biopsy samples from healthy tissue of colorectal carcinoma patients (CRC) were obtained in collaboration with the department of general surgery of the University Medicine Rostock, Germany. Sample details are provided in **Supplementary Table S9.**

*Healthy human blood:* Samples were collected from 18 volunteer workers at the University Hospital Schleswig-Holstein, Kiel, Germany (**Supplementary Table S8**).

The study was approved by the ethics committee of the medical faculty of the Christian-Albrecht University of Kiel, Kiel, Germany. All study participants signed a written consent.

## Human mononuclear cell staining for flow cytometry immunophenotyping

Mononuclear cells were washed with PBS + 2mM EDTA + 0.5% BSA (PEB buffer) and then stained with a cell surface antibody cocktail for CD3 -Alexa Fluor 700 (OKT3, Biolegend), CD4 APC-Vio700

(M-T466, MiltenyiBiotech), CD8 VioGreen (REA734, MiltenyiBiotech), CD14 PerCP (TÜK4, MiltenyiBiotech), CD20 PerCP (LT20, MiltenyiBiotech), CD161 Brilliant Violet 421 (DX12, BD Biosciences), IL-18Rα -APC (REA1095, MiltenyiBiotech), IL-18Rα -PE (REA1095, MiltenyiBiotech), TRAV12-1 FITC (F1, Invitrogen), TRAV1-2 PE-Vio700 (REA179, MiltenyiBiotech), MR1 5-OP-RU APC (NIH), at room temperature for 30 min in the dark. Cells were washed again and resuspended in 300uL of PEB buffer. Propidium Iodide (MiltenyiBiotech) was used to exclude dead cells. Data were acquired on an LSR Fortessa (BD Bioscience, San Jose, CA, USA). Flow cytometry data were analysed using FlowJo (Treestar, Ashland, OR, USA) software.

*Rosati et al. - A novel unconventional T cell population enriched in Crohn's disease*

**Supplementary Table S8: Immunophenotyping blood sample collection phenotype information.** Information about disease phenotype was provided via de Montreal classification. Disease score was available through the Harvey-Bradshaw index. NA corresponds to "information not available".

| | Sample group | | |
|---|---|---|---|
| | **CD** | **UC** | **Healthy** |
| **Basic phenotype** | | | |
| Samples | 25 | 13 | 18 |
| Gender, M/F (%) | 14/11 (56/44%) | 8/5 (60/40%) | 8/10 (45/55%) |
| Age, mean±std | 43±16 | 43±12 | 31±8 |
| **Montreal classification** | | | |
| *Age at diagnosis in years* | | | |
| A1, <16 (%) | 4 (16%) | – | – |
| A2, 17-40 (%) | 13 (52%) | – | – |
| A3, >40 (%) | 4 (20%) | – | – |
| Info not available (NA) | 4 | | |
| *Behaviour* | | | |
| B1, inflammatory (%) | 9 (36%) | – | – |
| B2, stricturing (%) | 6 (24%) | – | – |
| B3, penetrating (%) | 7 (28%) | – | – |
| Info not available (NA) | 3 | – | – |
| *Location* | | | |
| L1, ileal (%) | 11 (44%) | – | – |
| L2, colonic (%) | 1 (4%) | – | – |
| L3, ileocolonic (%) | 9 (36%) | – | – |
| Info not available (NA) | 4 | – | – |
| *Severity* | | | |
| S0, Clinical remission | – | 5 (41%) | – |
| S1, Mild | – | 2 (16%) | – |
| S2 Moderate | – | – | – |
| S3 Severe | – | – | – |
| Info not available (NA) | | 6 | – |
| *Extensity* | | | |
| E1 Ulcerative proctitis | – | – | – |
| E2 Left-side UC | – | 4 (33%) | – |
| E3 Extensive | – | 3 (25%) | – |
| Info not available (NA) | – | 6 | |
| **Disease activity** | | | |
| HBI, mean±std | 3 ±3 | – | – |
| Clinical remission, HBI≤4 (%) | 10 (40%) | | |
| Mild activity, 5≤HBI≤7 (%) | 6 (24%) | | – |
| Moderate activity, 8≤HBI≤16 (%) | 1 (4%) | – | – |
| Info not available (NA) | 8 | – | – |

*Rosati et al. - A novel unconventional T cell population enriched in Crohn's disease*

**Supplementary Table S9: Immunophenotyping blood and intestinal tissue sample collection phenotype information.** Information about disease phenotype was provided via de Montreal classification. Disease score was available through the Harvey-Bradshaw index. NA corresponds to "information not available".

| | Sample group | | |
|---|---|---|---|
| | **CD** | **UC** | **CRC** |
| **Basic phenotype** | | | |
| Samples | 14 | 7 | 19 |
| Gender, M/F (%) | 7/7 (50/50%) | 6/1 (85/15%) | 11/8 (57/43%) |
| Age, mean±std | 44±18 | 31±8 | 68±14 |
| **Tissue** | | | |
| Blood | 14 | 7 | 0 |
| Gut (colon biopsy) | 14 | 7 | 19 |
| **Montreal classification** | | | |
| *Age at diagnosis in years* | | | |
| A1, <16) (%) | – | – | – |
| A2, 17-40 (%) | – | – | – |
| A3, >40 (%) | – | – | – |
| Info not available (NA) | 14 | | |
| *Behaviour* | | | |
| B1, inflammatory (%) | 7 (50%) | – | – |
| B2, stricturing (%) | 2 (14%) | – | – |
| B3, penetrating (%) | 1 (7%) | – | – |
| Info not available (NA) | 4 | – | – |
| *Location* | | | |
| L1, ileal (%) | 5 (35%) | – | – |
| L2, colonic (%) | 3 (21%) | – | – |
| L3, ileocolonic (%) | 1 (7%) | – | – |
| Info not available (NA) | 5 | – | – |
| *Severity* | | | |
| S0, Clinical remission | – | – | – |
| S1, Mild | – | 1 (14%) | – |
| S2, Moderate | – | 3 (42%) | – |
| S3, Severe | – | – | – |
| Info not available (NA) | – | 3 | – |
| *Extensity* | | | |
| E1 Ulcerative proctitis | – | 3 (42%) | – |
| E2 Left-side UC | – | 2 (28%) | – |
| E3 Extensive | – | – | – |
| Info not available (NA) | – | 2 | – |
| **Disease activity** | | | |
| Mild activity, 5≤HBI≤7 (%) | 1 (7%) | 3 (42%) | – |
| Moderate activity, 8≤HBI≤16 (%) | – | 2 (28%) | – |
| Severe activity, 16≤HBI≤∞ (%) | – | 1 (14%) | – |
| Info not available (NA) | 13 | 1 | – |

## References


1. R. F. Harvey, J. M. Bradshaw, A SIMPLE INDEX OF CROHN'S-DISEASE ACTIVITY. *The Lancet* **315**, 514-514 (1980).
2. S. Waniek *et al.*, Association of Vitamin E Levels with Metabolic Syndrome, and MRI-Derived Body Fat Volumes and Liver Fat Content. *Nutrients* **9**, (2017).
3. M. Koch *et al.*, Dietary patterns associated with magnetic resonance imaging–determined liver fat content in a general population study. *The American Journal of Clinical Nutrition* **99**, 369-377 (2013).
4. U. Nöthlings, M. Krawczak, PopGen. *Bundesgesundheitsblatt - Gesundheitsforschung - Gesundheitsschutz* **55**, 831-835 (2012).
5. F. Degenhardt *et al.*, Construction and benchmarking of a multi-ethnic reference panel for the imputation of HLA class I and II alleles. *Human Molecular Genetics* **28**, 2078-2092 (2018).
6. X. Zheng *et al.*, HIBAG—HLA genotype imputation with attribute bagging. *The Pharmacogenomics Journal* **14**, 192-200 (2014).
7. F. Degenhardt *et al.*, Trans-ethnic analysis of the human leukocyte antigen region for ulcerative colitis reveals shared but also ethnicity-specific disease associations. *Hum Mol Genet*, (2021).
8. M. V. Pogorelyy *et al.*, Persisting fetal clonotypes influence the structure and overlap of adult human T cell receptor repertoires. *PLOS Computational Biology* **13**, e1005572-e1005572 (2017).
9. M. Shugay *et al.*, Towards error-free profiling of immune repertoires. *Nature Methods* **11**, 653-655 (2014).
10. D. a. Bolotin *et al.*, MiXCR: software for comprehensive adaptive immunity profiling. *Nature Methods* **12**, 380-381 (2015).
11. Y. Benjamini, Y. Hochberg. (WileyRoyal Statistical Society, 1995), vol. 57, pp. 289-300.
12. N. T. Csardi Gabor, The igraph software package for complex network research. *InterJournal, Complex Systems* **5**, 1–9 (2006).
13. R. Gowthaman, B. G. Pierce, TCRmodel: high resolution modeling of T cell receptors from sequence. *Nucleic Acids Res* **46**, W396-w401 (2018).
14. A. Butler, P. Hoffman, P. Smibert, E. Papalexi, R. Satija, Integrating single-cell transcriptomic data across different conditions, technologies, and species. *Nature Biotechnology* **36**, 411-420 (2018).
15. I. Korsunsky *et al.*, Fast, sensitive and accurate integration of single-cell data with Harmony. *Nature Methods* **16**, 1289-1296 (2019).
16. G. Finak *et al.*, MAST: a flexible statistical framework for assessing transcriptional changes and characterizing heterogeneity in single-cell RNA sequencing data. *Genome biology* **16**, 278-278 (2015).

*Rosati et al. - A novel unconventional T cell population enriched in Crohn's disease*

## Supplementary Figures

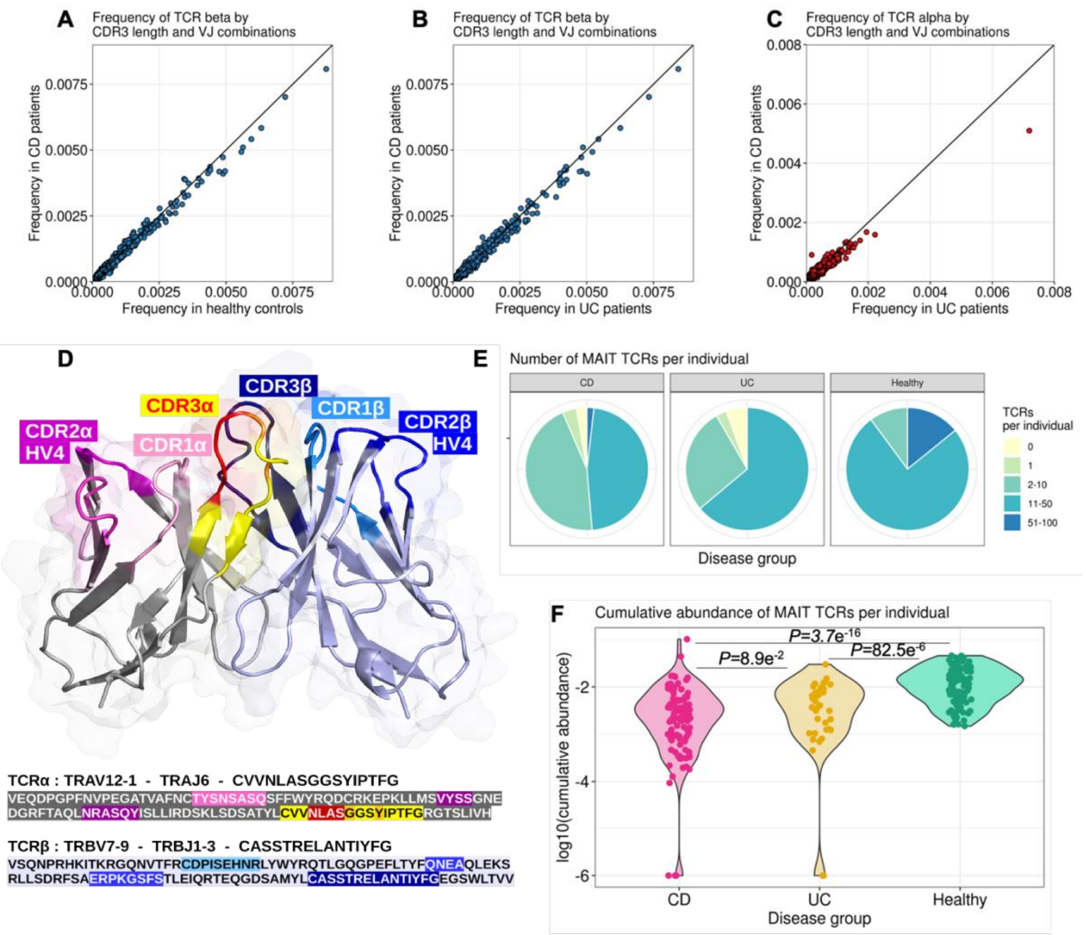


**Supplementary Figure S1: TCR groups comparison among disease groups, representative CAIT TCR structure and presence and abundance of MAIT clonotypes in the whole blood collection**. **(A)** Scatterplot of the frequency of all CDR3 lengths and VJ gene combinations in CD patients and healthy controls for TCR beta. **(B)** UC patients and CD patients for TCR beta **(C)** UC patients and CD patients for TCR alpha. **(D)** Protein structural model of the TCR receptor TRAV12-1-TRAJ6-CVVNLASGGSYIPTF / TRBV7-9-TRBJ1-3-CASSTRELANTIYF. CDR1, CDR2, CDR3 and CDR2-HV4 loops are highlighted as indicated in boxes and colors as indicated in the sequence panel below. TCRα non-CDR regions are colored grey, TCRβ in light blue. **(E)** Pie chart showing the proportion of individuals carrying different numbers of MAIT clonotypes. **(F)** Cumulative abundance of MAIT clonotypes per individual. Differences between disease groups have been assessed using Mann-Whitney U test, followed by FDR multiple comparison correction.

*Rosati et al. - A novel unconventional T cell population enriched in Crohn's disease*

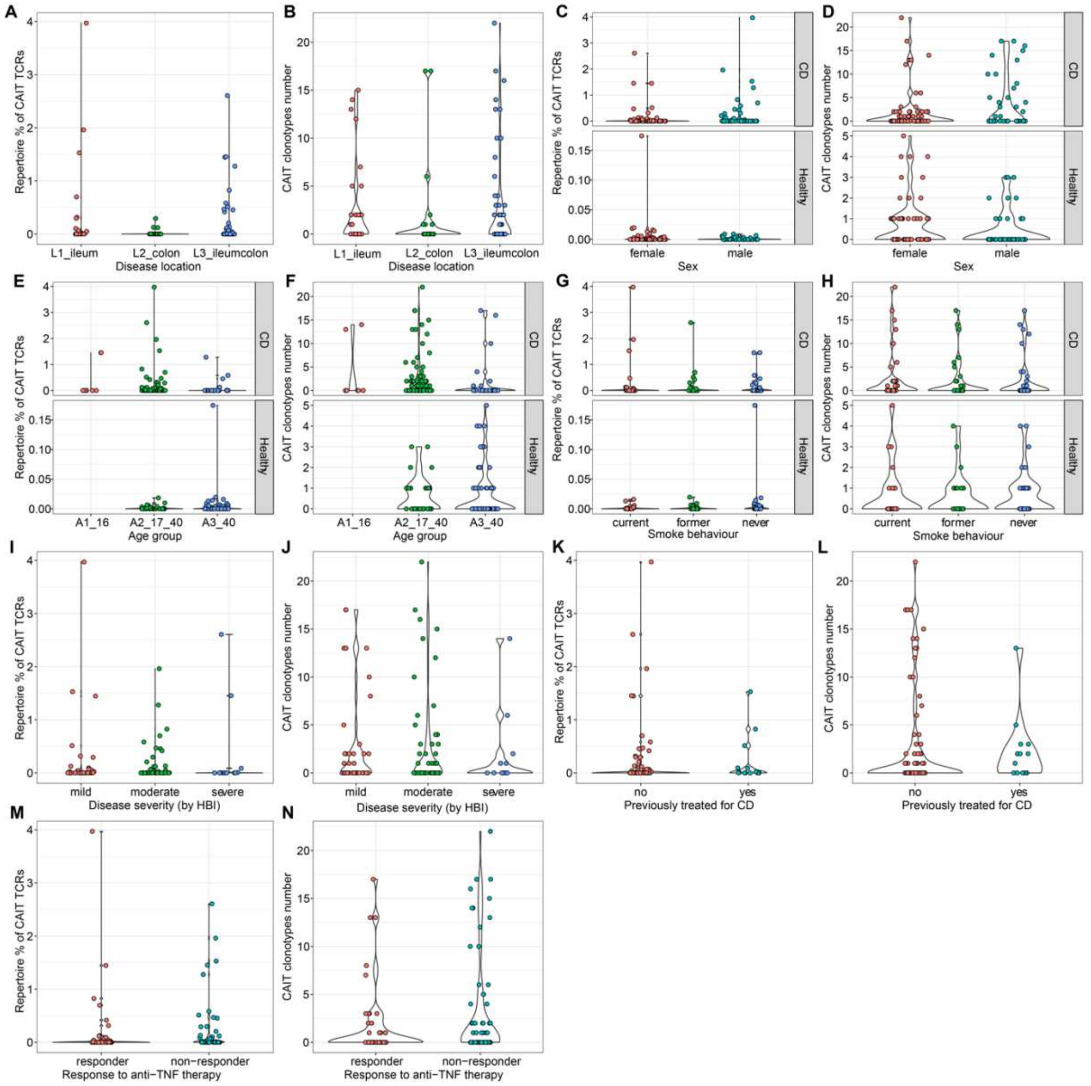


**Supplementary Figure S2: Correlation of CAIT clonotypes with phenotype and clinical parameters. (A)** CAIT repertoire % and disease location as for the Montreal classification in CD patients **(B)** Number of CAIT clonotypes and disease location as for the Montreal classification in CD patients **(C)** CAIT repertoire % and sex for CD patients and healthy controls **(D)** Number of CAIT clonotypes and sex for CD patients and healthy controls **(E)** CAIT repertoire % and age groups as for the Montreal classification, for CD patients and healthy controls **(F)** Number of CAIT clonotypes and age groups as for the Montreal classification, for CD patients and healthy controls **(G)** CAIT repertoire % and smoking behaviour, for CD patients and healthy controls **(H)** Number of CAIT clonotypes and smoking behaviour, for CD patients and healthy controls **(I)** CAIT repertoire % and disease activity as per the Harvey-Bradshaw index (HBI). Mild: 5≤HBI≤7. Moderate: 8≤HBI≤15. Severe: 16≤HBI≤∞, for CD patients. **(J)** Number of CAIT clonotypes and disease activity as per the Harvey-Bradshaw index (HBI). Mild: 5≤HBI≤7. Moderate: 8≤HBI≤15. Severe: 16≤HBI≤∞, for CD patients. **(K)** CAIT repertoire % and status of previous treatment for CD (treated/yes and untreated/no). **(L)** Number of CAIT clonotypes status and of previous treatment for CD (treated/yes and untreated/no). **(M)** CAIT repertoire % at baseline and response to anti-TNF treatment, in CD patients. **(N)** Number of CAIT clonotypes at baseline and response to anti-TNF treatment, in CD patients. Differences between disease groups have been assessed using Mann-Whitney U test, followed by FDR multiple comparison correction.

*Rosati et al. - A novel unconventional T cell population enriched in Crohn's disease*

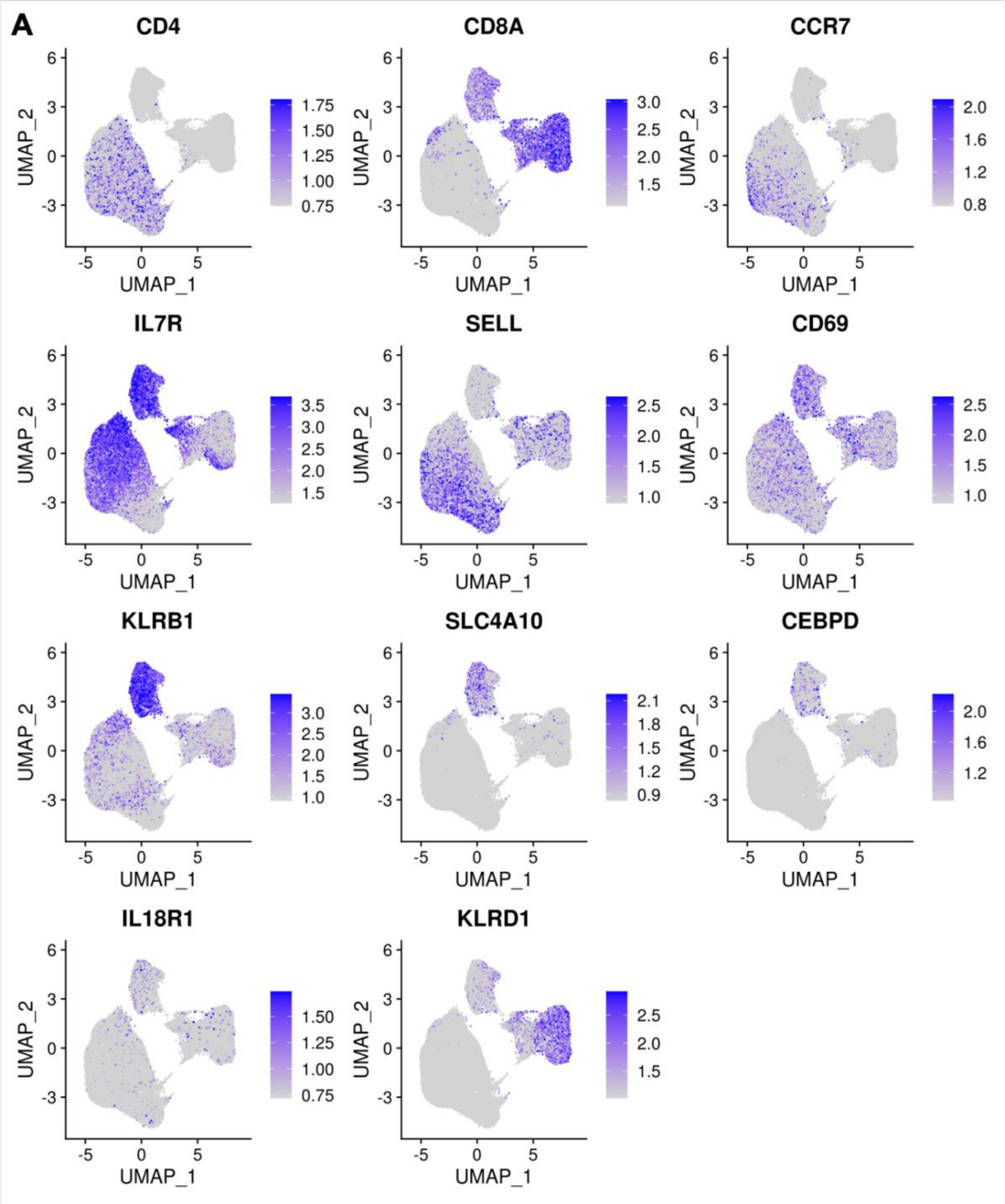


**Supplementary Figure S3: Distribution of Seurat cluster marker genes in single-cell data (A)** Distribution of marker genes among single-cell UMAP plot.

*Rosati et al. - A novel unconventional T cell population enriched in Crohn's disease*

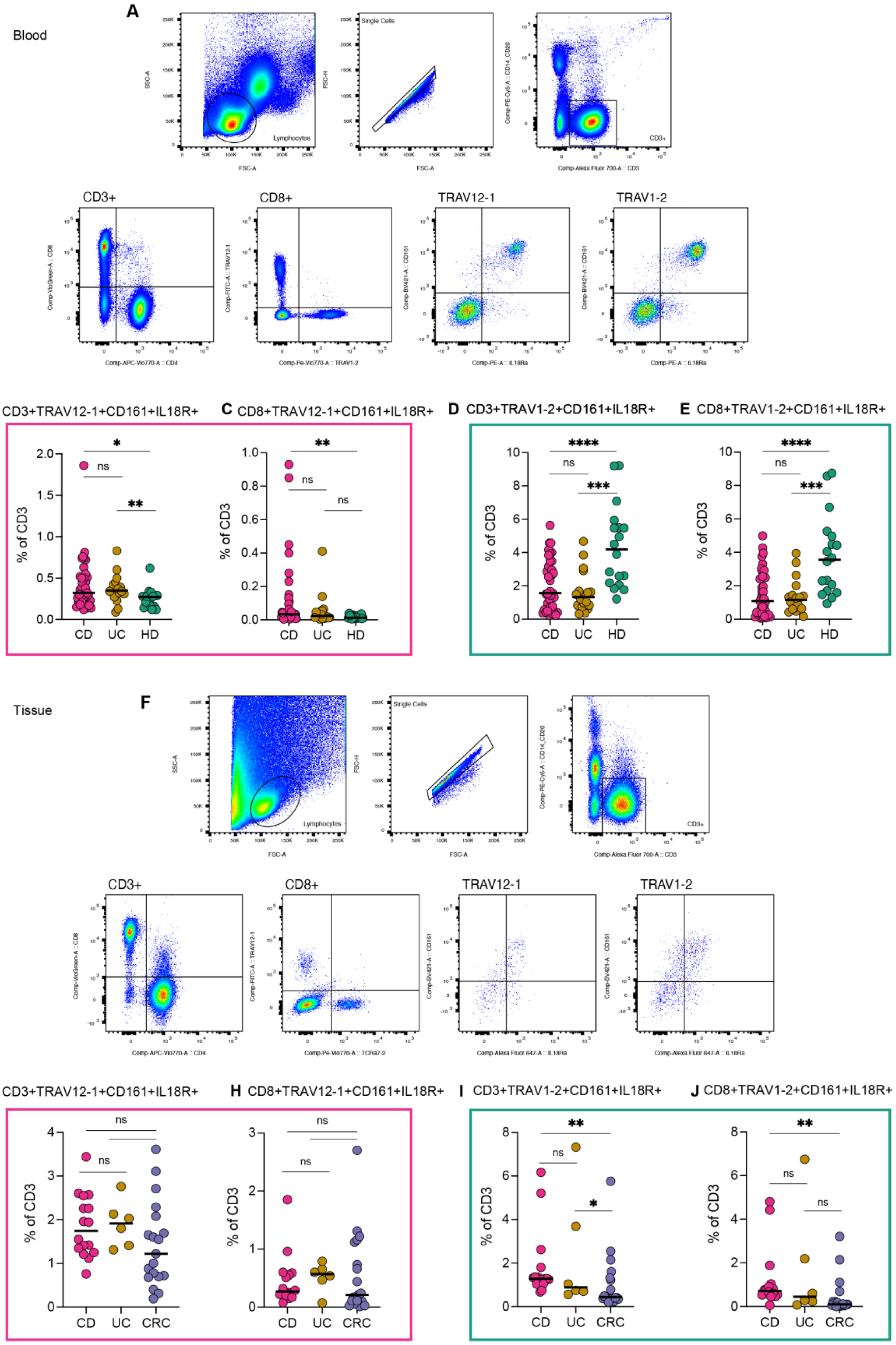

*Rosati et al. - A novel unconventional T cell population enriched in Crohn's disease*

**Supplementary figure S4: Immunophenotyping of TRAV1-2⁺ or TRAV12-1⁺ CD161⁺IL18R⁺ cells. (A)** Flow cytometry plots showing gating strategy fort the immunophenotyping analysis done in peripheral blood from one representative CD patient. All stainings were performed on freshly isolated PBMCs. After exclusion of doublets and dead cells, all analyses were performed on CD14-CD20-CD3+ lymphocytes. Stains of CD4 and CD8 cells were used to investigate subpopulations of CAIT cells.

(B) PBMCs were gated on CD3+ lymphocytes and the percentage of $TRAV12\text{-}1^{+}CD161^{+}IL18R^{+}$ cells (which include CAIT cells) among CD3+ is indicated.

(C) PBMCs were gated on CD3+CD8+ lymphocytes and the percentage $TRAV12\text{-}1^{+}CD161^{+}IL18R^{+}$ (which include CAIT cells) cells among CD3+ is indicated.

(D) PBMCs were gated on CD3+ lymphocytes and the percentage of $TRAV1\text{-}2^{+}CD161^{+}IL18R^{+}$ cells (which include MAIT cells) among CD3+ is indicated.

(E) PBMCs were gated on CD3+CD8+ lymphocytes and the percentage $TRAV1\text{-}2^{+}CD161^{+}IL18R^{+}$ cells (which include MAIT cells) among CD3+ is indicated.

**(F)** Flow cytometry plots of cells isolated from intestinal mucosa of one representative CD patient.

(G) Intestinal cells were gated on CD3+ lymphocytes and the percentage of $TRAV12\text{-}1^{+}CD161^{+}IL18R^{+}$ cells (which include CAIT cells) among CD3+ is indicated.

(H) Intestinal cells were gated on CD3+CD8+ lymphocytes and the percentage $TRAV12\text{-}1^{+}CD161^{+}IL18R^{+}$ (which include CAIT cells) cells among CD3+ is indicated.

(I) Intestinal cells were gated on CD3+ lymphocytes and the percentage of $TRAV1\text{-}2^{+}CD161^{+}IL18R^{+}$ cells (which include MAIT cells) among CD3+ is indicated.

(J) Intestinal cells were gated on CD3+CD8+ lymphocytes and the percentage $TRAV1\text{-}2^{+}CD161^{+}IL18R^{+}$ cells (which include MAIT cells) among CD3+ is indicated.

Sample size of peripheral blood samples was n=39 CD patients, n=19 UC patients and n=18 healthy controls. For matched samples of blood and intestinal biopsies there were n=14 CD patients, n=7 UC patients and n=19 CRC control patients. Mann-Whitney U test, ns: $P > 0.05$, * $P \le 0.05$, ** $P \le 0.01$, *** $P \le 0.001$, **** $P \le 0.0001$.
